# [^212^Pb]Pb-eSOMA-01: A Promising Radioligand for Targeted Alpha Therapy of Neuroendocrine Tumors

**DOI:** 10.3390/ph16070985

**Published:** 2023-07-10

**Authors:** Dylan Chapeau, Sofia Koustoulidou, Maryana Handula, Savanne Beekman, Corrina de Ridder, Debra Stuurman, Erik de Blois, Yulia Buchatskaya, Karlijn van der Schilden, Marion de Jong, Mark W. Konijnenberg, Yann Seimbille

**Affiliations:** 1 Erasmus MC, Department of Radiology and Nuclear Medicine, University Medical Center Rotterdam, 3015 GD Rotterdam, The Netherlands; d.chapeau@eramusmc.nl (D.C.); sofiakoustoulidou@gmail.com (S.K.); m.handula@erasmusmc.nl (M.H.); s.beekman@erasmusmc.nl (S.B.); c.deridder@erasmusmc.nl (C.d.R.); d.stuurman@erasmusmc.nl (D.S.); r.deblois@erasmusmc.nl (E.d.B.); m.hendriks-dejong@erasmusmc.nl (M.d.J.); m.konijnenberg@erasmusmc.nl (M.W.K.); 2 Erasmus MC Cancer Institute, 3015 GD Rotterdam, The Netherlands; 3 Nuclear Research & Consultancy Group, 1755 LE Petten, The Netherlands; buchatskaya@nrg.eu (Y.B.); k.vanderschilden@nrg.eu (K.v.d.S.); 4 TRIUMF, Life Sciences Division, Vancouver, BC V6T 2A3, Canada

**Keywords:** NETs, SSTR2, targeted alpha therapy, ^203^Pb, ^212^Pb

## Abstract

Peptide receptor radionuclide therapy (PRRT) has been applied to the treatment of neuroendocrine tumors (NETs) for over two decades. However, improvement is still needed, and targeted alpha therapy (TAT) with alpha emitters such as lead-212 (^212^Pb) represents a promising avenue. A series of ligands based on octreotate was developed. Lead-203 was used as an imaging surrogate for the selection of the best candidate for the studies with lead-212. ^203/212^Pb radiolabeling and in vitro assays were carried out, followed by SPECT/CT imaging and ex vivo biodistribution in NCI-H69 tumor-bearing mice. High radiochemical yields (≥99%) and purity (≥96%) were obtained for all ligands. [^203^Pb]Pb-eSOMA-01 and [^203^Pb]Pb-eSOMA-02 showed high stability in PBS and mouse serum up to 24 h, whereas [^203^Pb]Pb-eSOMA-03 was unstable in those conditions. All compounds exhibited a nanomolar affinity (2.5–3.1 nM) for SSTR2. SPECT/CT images revealed high tumor uptake at 1, 4, and 24 h post-injection of [^203^Pb]Pb-eSOMA-01/02. Ex vivo biodistribution studies confirmed that the highest uptake in tumors was observed with [^212^Pb]Pb-eSOMA-01. [^212^Pb]Pb-eESOMA-01 displayed the highest absorbed dose in the tumor (35.49 Gy/MBq) and the lowest absorbed dose in the kidneys (121.73 Gy/MBq) among the three tested radioligands. [^212^Pb]Pb-eSOMA-01 is a promising candidate for targeted alpha therapy of NETs. Further investigations are required to confirm its potential.

## 1. Introduction

Neuroendocrine tumors (NETs) are a rare class of tumors arising from neuroendocrine cells. NETs occur throughout the body, although the majority arise in the gastroenteropancreatic (GEP) tract or the lung. Surgical resection is the standard of care for localized NETs, while chemotherapy and targeted drug therapy are often administered to patients with metastatic disease. However, the slow-growing nature of NETs makes these treatments relatively inefficient. PRRT (Peptide receptor radionuclide therapy) represents an interesting therapeutic option for advanced metastatic NET patients. PRRT is a molecularly targeted therapy that makes use of a peptide linked to a radionuclide emitting ionizing radiation to induce tumor cell death. For NETs, the peptide is designed to bind selectively to the somatostatin receptor subtype 2 (SSTR2), which is highly upregulated in neuroendocrine tumor cells.

The first radiopeptide used for PRRT of NETs was [^111^In]In-DTPA-octreotide (Octreoscan^®^). Octreotide (TOC) was chosen because this peptide was found to bind selectively to SSTR2 [1]. Indium-111 was used as an Auger emitter, although it is commonly used for gamma imaging. Octreoscan^®^ demonstrated therapeutic efficacy without significant toxicity due to the high linear energy transfer (LET) of indium-111 [2,3,4]. However, the short range of the auger electrons hampered its efficacy in large tumors and end-stage patients. Therefore, a new generation of radioligands, labeled with beta emitters, was developed. The two most common beta-emitting radionuclides in PRRT are yttrium-90 (*T*_1/2_ = 64.05 h, E_βmax_ = 2.28 MeV) and lutetium-177 (*T*_1/2_ = 159.5 h, E_βmax_ = 0.50 MeV). Two SSTR2-targeting peptides labeled with these radionuclides, [^90^Y]Y-DOTA-TOC and [^177^Lu]Lu-DOTA-TATE, have been extensively studied over the last two decades [5,6,7,8]. Similar to octreotide, octreotate (TATE) was designed to target SSTR2 [1]. Both radiopharmaceuticals showed encouraging clinical results by improving progression-free survival and the quality of life of NET patients. [^177^Lu]Lu-DOTA-TATE (Lutathera^®^) was approved by the European Medicines Agency (EMA) and the Food and Drug Administration (FDA) in 2017 and 2018, respectively, for the treatment of GEP-NETs. However, the current PRRT of NETs requires improvement since the overall response rates remain insufficient and relapse often occurs 2–3 years after the first treatment [9].

The replacement of the conventional beta emitter by an alpha emitter has been recently explored to enhance the efficacy of PRRT. Only a few alpha-emitting radionuclides are considered suitable for targeted alpha therapy (TAT), such as actinum-225 (^225^Ac), bismuth-213 (^213^Bi), astatine-211 (^211^At), radium-223 (^223^Ra), and lead-212 (^212^Pb). The high LET of alpha particles (100 keV/µm) and their short range (50–100 µm) make them efficient in causing irreversible lethal damage to cancer cells via DNA double-strand breaks. Moreover, alpha particles induce less toxicity to neighboring normal organs compared to beta emitters. TAT of NETs using SSTR2-targeting ligands labeled with ^225^Ac, ^213^Bi, and ^212^Pb has already been reported and showed better therapeutic efficacy than conventional PRRT [10,11]. Lead-212 (^212^Pb; T_1/2_ = 10.6 h, E_βmax_ = 0.57 MeV) is a beta emitter that generates alpha particle radiation via its short-lived progenies ^212^Bi (*T*_1/2_ = 60.6 min, E_α_ = 6.1 MeV (35.9%), and E_βmax_ = 2.25 MeV (64.1%)) and ^212^Po (*T*_1/2_ = 0.3 µs, E_α_ = 8.8 MeV). 2,2′,2″,2″‘-(1,4,7,10-Tetraazacyclododecane-1,4,7,10-tetrayl)tetraacetic acid (DOTA)-chelator forms stable complexes with ^212^Pb, but one third of the resulting ^212^Bi can be lost from the DOTA chelator during ^212^Pb decay [12]. Furthermore, acid-catalyzed dissociation of ^212^Pb from DOTA conjugates after internalization has been reported as a source of bone marrow toxicity. Released ^212^Pb is transported to the bone, where it subsequently decays to ^212^Bi [13]. Replacement of the carboxylic acid donor arms of DOTA with amide groups yielded 1,4,7,10-tetraaza-1,4,7,10-tetra (2-carbamoylmethyl)cyclododecane (DOTAM), which is to date the best chelator for lead radionuclides. The complex of lead cation and DOTAM is less labile to metal ion release under lower pH conditions, conferring enhanced resistance to acid-catalyzed dissociation within the cell [14]. A recent clinical trial has demonstrated that no acute or late toxicity occurred in patients with ovarian cancer treated with [^212^Pb]Pb-TCMC-trastuzumab [15]. TAT with [^212^Pb]Pb-DOTAM-TATE has also shown a favorable safety profile in patients with neuroendocrine tumors [16].

**Figure 1:**
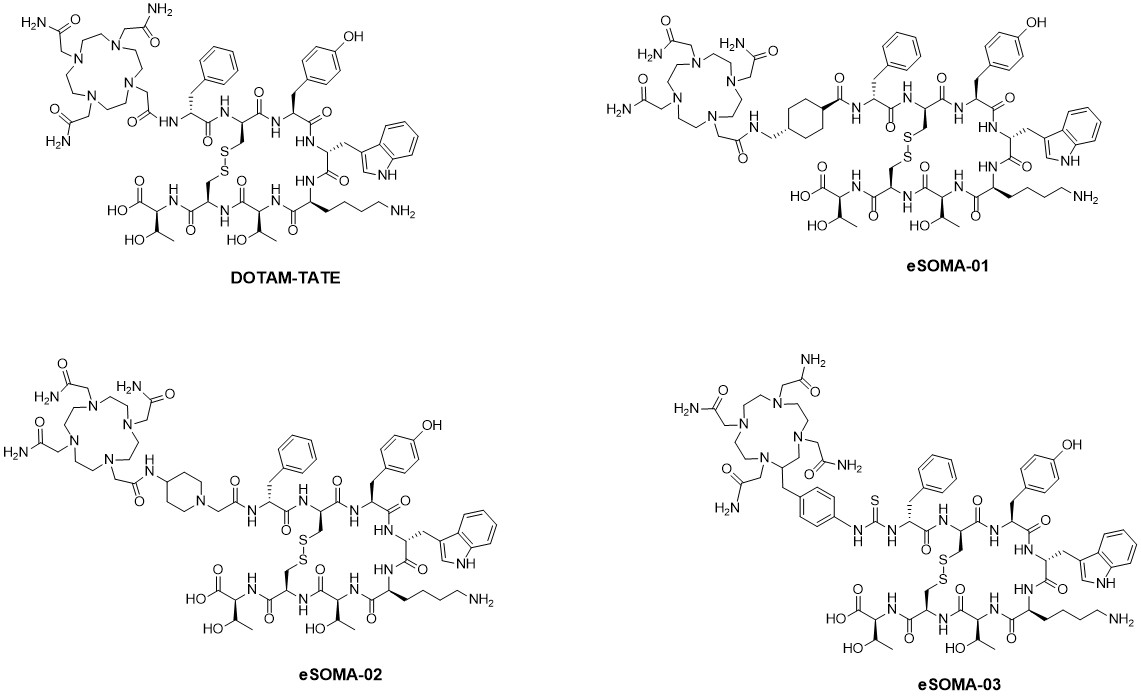
Chemical structures of DOTAM-TATE, eSOMA-01, eSOMA-02, and eSOMA-03.

Herein, we report the development of a new series of octreotate derivatives, dubbed eSOMA, containing either the DO3AM or *p*-Bn-SCN-TCMC chelator, two functionalized DOTAM derivatives, and an Amcha or Pip linker (Figure 1). The SSTR2-targeting ligands have been labeled with ^212^Pb and ^203^Pb, an imaging surrogate of lead-212. In vitro assays and non-invasive SPECT/CT scans were performed with the ^203^Pb-labeled analogs to determine the best candidate for further studies. Ex vivo biodistribution studies with the ^212^Pb-labeled lead compound were carried out to determine the potential of the eSOMA derivatives for targeted alpha therapy of NETs.

## 2. Results

### 2.1. Chemistry and Radiochemistry

**Scheme 1:**
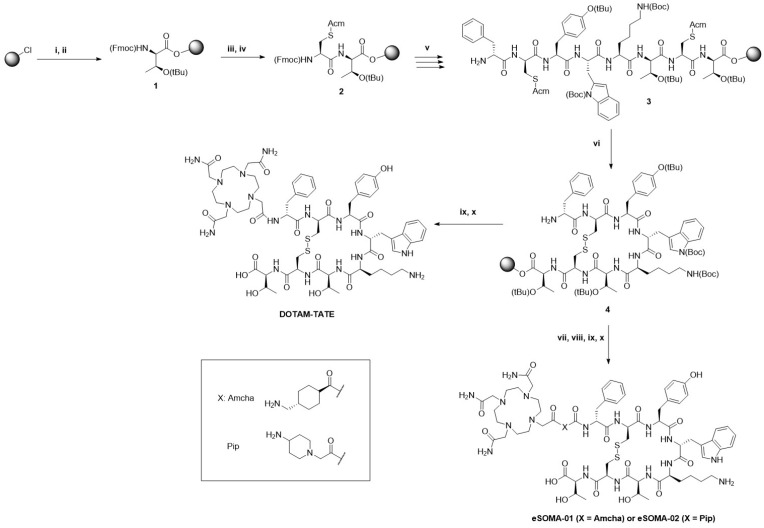
Synthesis of DOTAM-TATE, eSOMA-01, and eSOMA-02: (i) Fmoc-Thr(*t*Bu)-OH, DCM, DIPEA, 1 h 30 min, rt; (ii) DCM/MeOH/DIPEA (80:15:5 *v/v/v*), 15 min, rt; (iii) 20% 4-Methylpiperidine in DMF, 15 min, rt; (iv) Fmoc-Cys(Acm)-OH, HATU, DIPEA, 1 h, rt; (v) Elongation; (vi) Tl(TFA)_3_, DMF, 1 h, rt; (vii) 4-(Fmoc-aminomethyl)cyclohexanecarboxylic acid (Fmoc-Amcha-OH) or Fmoc-4-amino-1-carboxymethyl-piperidine (Fmoc-Pip-OH), HATU, DIPEA, 1 h, rt; (viii) 20% 4-Methylpiperidine in DMF, 15 min, rt; (ix) DO3AM acetic acid, PyBop, DIPEA, overnight, rt; (x) TFA/H_2_O/TIPS (95:2.5:25), 2 h, rt., 11% (DOTAM-TATE), 12% (eSOMA-01), and 7% (esOMA-02).

**Scheme 2:**
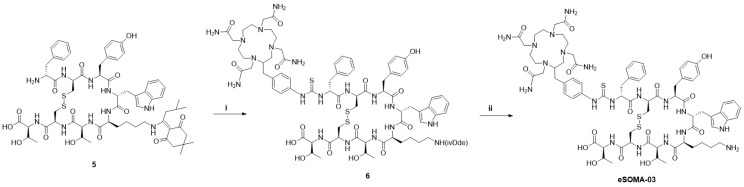
Synthesis of eSOMA-03: (i) *p*-SCN-Bn-TCMC, PyBop, DIPEA, overnight, rt, 17%; (ii) 2% hydrazine in DMF, 1 h, rt, 17%.

The synthesis of Tyr^3^-octreotate was performed on solid support using a standard Fmoc-based strategy (Scheme 1). The amino acids were coupled to each other using conventional coupling reagents, such as HATU. On-resin cyclization was carried out by treating the linear peptidyl-resin with Tl (TFA)_3_ to generate the intramolecular disulfide bond. The Acm-protecting group was specifically chosen for the cysteine residues because it can be selectively deprotected during the cyclization step [17]. Analysis of an aliquot demonstrated that the cyclic-protected d-Phe-cyclo[Cys-Tyr(*t*Bu)-d-Trp(Boc)-Lys(Boc)-Thr(*t*Bu)-Cys]-Thr(*t*Bu)-resin (4) was obtained in quantitative yield. DOTAM-TATE was synthesized by coupling DO3AM acetic acid to 4 in the presence of PyBOP, followed by the deprotection of the side-chain protecting groups and cleavage from the solid support. DOTAM-TATE was obtained with a yield of 11% after semi-preparative HPLC purification. eSOMA-01 and eSOMA-02 were obtained via the same approach after attachment of the corresponding linkers (Amcha or Pip) to the resin-bound protected Tyr^3^-octreotate 4 and removal of the Fmoc group. eSOMA-01 and eSOMA-02 were obtained with 12% and 7% yields, respectively, after purification. Synthesis of d-Phe-Cys(Acm)-Tyr(*t*Bu)-d-Trp(Boc)-Lys(ivDde)-Thr(*t*Bu)-Cys(Acm)-Thr(*t*Bu)-resin, containing a lysine residue protected with an ivDde instead of the Boc protecting group used for 4, was required to obtain eSOMA-03 (Scheme 2). After elongation and cyclization, the peptide was cleaved from the resin and coupled to *p*-SCN-Bn-TCMC to yield intermediate 6. Finally, deprotection of the ivDde group and semi-preparative HPLC purification afforded eSOMA-03 in 17% yield.

**Figure 2:**
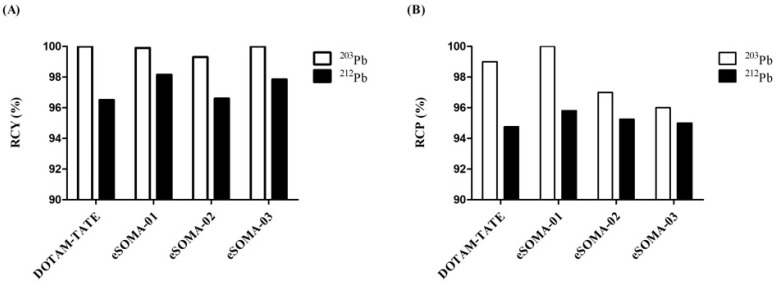
RCY (**A**) and RCP (**B**) obtained from the labeling of DOTAM-TATE, eSOMA-01, eSOMA-02, and eSOMA-03 with ^203^Pb and ^212^Pb.

Labeling of DOTAM-TATE, eSOMA-01, eSOMA-02, and eSOMA-03 was performed with ^203/212^PbCl_2_ at room temperature in the presence of sodium acetate buffer and a mixture of ascorbic acid and gentisic acid to prevent radiolysis. The radiochemical yield (RCY) and radiochemical purity (RCP) were determined by iTLC and radio-HPLC, respectively (Figure 2). All labeled compounds were obtained with a RCY ≥ 96% and a RCP ≥ 95%.

### 2.2. In Vitro Characterization of DOTAM-TATE, eSOMA-01, eSOMA-02, and eSOMA-03

LogD_7_._4_ of the four ligands were all negative, confirming their hydrophilic nature (Table 1). Stability studies in PBS buffer and mouse serum demonstrated the high stability of [^203^Pb]Pb-DOTAM-TATE, [^203^Pb]Pb-eSOMA-01, and [^203^Pb]Pb-eSOMA-02. These radioligands exhibited stability over 97% in both media at 24 h. However, [^203^Pb]Pb-eSOMA-03 was relatively unstable in PBS (70% and 15% of intact peptide at 1 and 24 h, respectively), as well as in mouse serum (85% and 60% of intact peptide at 1 and 24 h, respectively). High stability of the corresponding ^212^Pb-labeled analogs was observed for [^212^Pb]Pb-DOTAM-TATE, [^212^Pb]Pb-eSOMA-01, and [^212^Pb]Pb-eSOMA-02 in PBS buffer and mouse serum (Table 2).

IC_50_ values were obtained by a competitive binding assay using [^111^In]In-DOTA-TATE as radioligand and purified Chinese hamster ovary-K1 (CHO-K1) membranes overexpressing the somatostatin receptor subtype 2 (Table 3). The four ligands exhibited nanomolar affinity for SSTR2. eSOMA-01, eSOMA-02, and eSOMA-03 showed IC_50_ values relatively similar to the parent peptide DOTA-TATE but 2.8- to 3.5-fold higher than the IC_50_ value of the reference DOTAM-TATE. However, after complexation with lead, the IC_50_ values of eSOMA-01, eSOMA-02, and eSOMA-03 were 1.1- to 1.5-fold lower than the IC_50_ value of [^nat^Pb]Pb-DOTAM-TATE.

### 2.3. In Vivo Preclinical Evaluation

**Figure 3:**
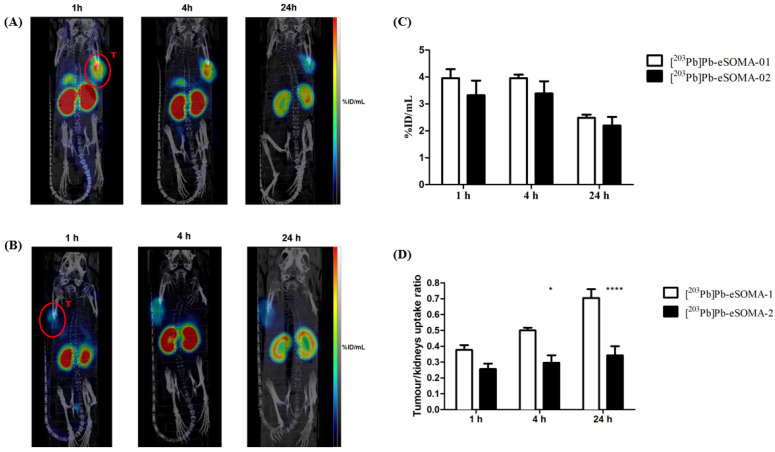
SPECT/CT scans of H69 tumor-bearing mice (*n* = 4) after administration of [^203^Pb]Pb-eSOMA-01 (**A**) and [^203^Pb]Pb-eSOMA-02 (**B**). Tumors were located on the right or left shoulder (red **T**). Tumor uptake is expressed as a percentage of injected dose per mL (% ID/mL) (**C**) and tumor/kidney ratios (**D**) derived from the SPECT images. * *p* < 0.05; **** *p* < 0.0001.

SPECT/CT imaging was performed in H69-xenografted Balb/c nu/nu mice at 1, 4, and 24 h after the administration of [^203^Pb]Pb-eSOMA-01 or [^203^Pb]Pb-eSOMA-02. Tumors were visible by SPECT/CT for both radioligands (Figure 3A,B). As expected, high uptake was observed in the kidneys as a result of the renal excretion of the radioligands and partial reabsorption in proximal tubule cells. Analysis of the SPECT images showed a similar tumor uptake (~3–4% ID/mL, *p* > 0.05) for both ^203^Pb-labeled compounds (Figure 3C). However, we noticed that the tumor/kidney ratios were significantly different (*p* < 0.05) from 4 h to 24 h between the two radioligands (Figure 3D). They increased over time for [^203^Pb]Pb-eSOMA-01 (0.5 ± 0.03 at 4 h to 0.70 ± 0.11 at 24 h), while they remained nearly constant for [^203^Pb]Pb-eSOMA-02 (0.30 ± 0.09 at 4 h and 0.4 ± 0.03 at 24 h).

**Figure 4:**
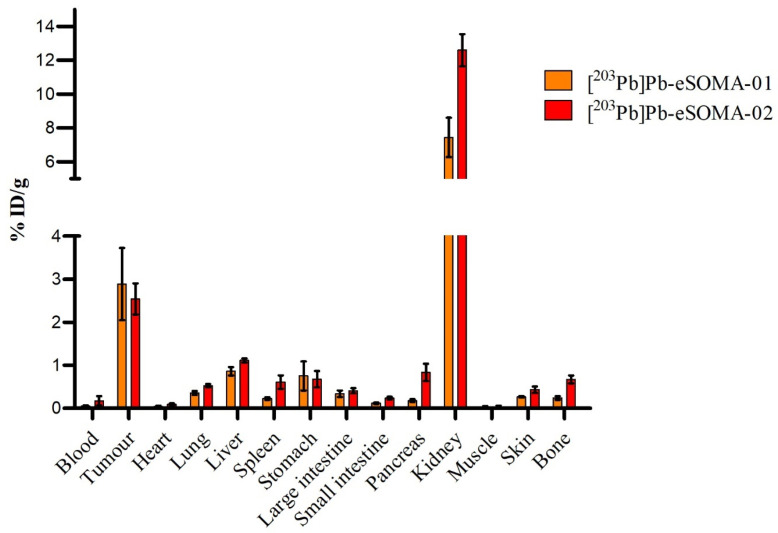
Results of the *ex-vivo* biodistribution studies performed 24 h after i.v. injection of [^203^Pb]Pb-eSOMA-01 and [^203^Pb]Pb-eSOMA-02 in H69 tumor bearing mice.

Ex vivo biodistribution performed 24 h post-injection of the ^203^Pb-labeled octreotate derivatives confirmed the results of the imaging studies (tumor uptake of ~3% ID/mL). The biodistribution profiles of [^203^Pb]Pb-eSOMA-01 and [^203^Pb]Pb-eSOMA-02 were relatively similar, but ^203^Pb-eSOMA-02 exhibited around 41% more uptake in the kidneys (*p* < 0.05) compared to [^203^Pb]Pb-eSOMA-01 after 4 h (Figure 4). Furthermore, [^203^Pb]Pb-eSOMA-01 showed a faster renal clearance than [^203^Pb]Pb-eSOMA-02 (half-life of 14.7 ± 2.4 h vs. 24.2 ± 4.5 h (*p* = 0.01) for [^203^Pb]Pb-eSOMA-01 and [^203^Pb]Pb-eSOMA-02, respectively). Uptakes in all the other non-target organs were below 1% ID/g for both radioligands, but slightly higher uptake in the spleen, pancreas, lung, liver, skin, and bone were observed for [^203^Pb]Pb-eSOMA-02 than [^203^Pb]Pb-eSOMA-01.

**Figure 5:**
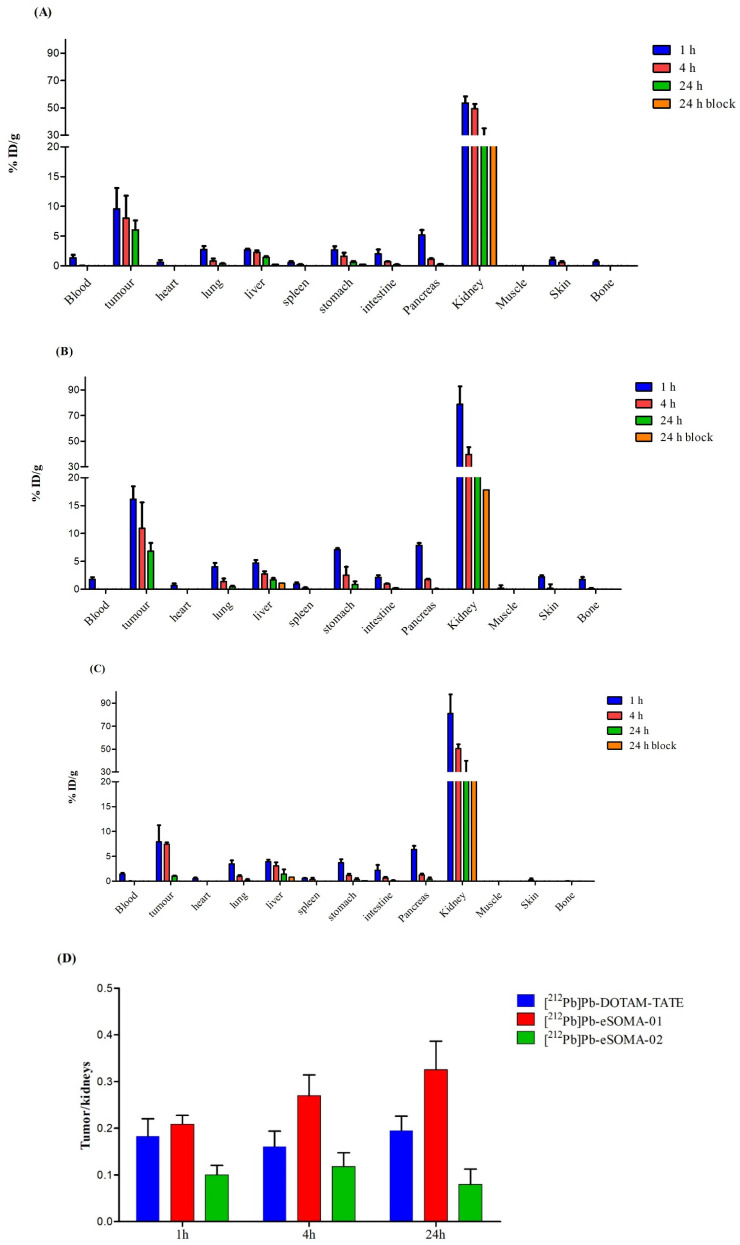
Biodistribution of [^212^Pb]Pb-DOTAM-TATE (**A**), [^212^Pb]Pb-eSOMA-01 (**B**), or [^212^Pb]Pb-eSOMA-02 (**C**) in H69-xenograft mice at 1, 4, and 24 h and the tumor-to-kidney ratios (**D**). Uptake in tissues is expressed as the percentage of the injected dose per gram of tissue (% ID/g).

**Figure 6:**
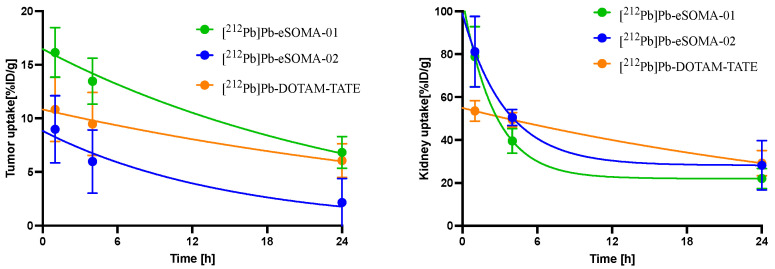
Kinetics of the tumor uptake (**left**) and the kidney uptake (**right**) of [^212^Pb]Pb-DOTAM-TATE, [^212^Pb]Pb-eSOMA-01, and [^212^Pb]Pb-eSOMA-02.

Biodistribution of [^212^Pb]Pb-DOTAM-TATE, [^212^Pb]Pb-eSOMA-01, and [^212^Pb]Pb-eSOMA-02 in H69-xenograft Balb/c nu/nu mice at 3 different time points is depicted in Figure 5. High tumor uptake was observed for all ^212^Pb-labeled compounds. [^212^Pb]Pb-eSOMA-01 exhibited 28% more tumor uptake at 1 h (*p* < 0.05) than the reference [^212^Pb]Pb-DOTAM-TATE and 61% more at 1 h and 24 h (*p* < 0.05) than [^212^Pb]Pb-eSOMA-02 (Tables S2–4). However, at 4 and 24 h post-injection, tumor uptake of [^212^Pb]Pb-DOTAM-TATE and [^212^Pb]Pb-eSOMA-01 was at the same level (~9 and 6% ID/g at 4 h and 24 h, respectively, *p* > 0.05). Consequently, clearance from the tumor was slower for [^212^Pb]Pb-eSOMA-01 than [^212^Pb]Pb-eSOMA-02 (half-life of 18.9 ± 6.1 h vs. 9.3 ± 4.2 h, respectively; NS), but faster than [^212^Pb]Pb-DOTAM-TATE (half-life of 37.1 ± 29.6 h) (Figure 6). Efficient blockage of the tumor uptake with co-injection of an excess of unlabeled ligand was observed at 24 h p.i. for all three radioligands, demonstrating specificity towards SSTR2. As for the ^203^Pb-labeled compounds, high uptake was found in the kidneys for all compounds. Clearance in the kidneys was faster for [^212^Pb]Pb-eSOMA-01 than for [^212^Pb]Pb-DOTAM-TATE and [^212^Pb]Pb-eSOMA-02 (half-life of 1.8 ± 0.5 h vs. 26.3 ± 3.8 h and 2.4 ± 1.0 h). [^212^Pb]Pb-eSOMA-01 showed higher uptake in the stomach at 1 h post-injection than the other radiotracers (7.1 vs. 2.7 and 3.7% ID/g for [^212^Pb]Pb-DOTAM-TATE and [^212^Pb]Pb-eSOMA-02, respectively, *p* < 0.05), while in the pancreas they all displayed a similar accumulation (~6 to 8% ID/g, *p* > 0.05). However, radioactivity was rapidly cleared from those organs. In the other non-target organs, the uptake was constantly below 5% ID/g at all time points. Furthermore, [^212^Pb]Pb-eSOMA-01 showed an increase in the tumor/kidney ratio over time (0.19 ± 0.03 at 1 h to 0.42 ± 0.10 at 24 h), whereas the tumor/kidney ratios remained constant for [^212^Pb]Pb-DOTAM-TATE (~0.20 ± 0.04) and reached a maximum at 4 h p.i. for [^212^Pb]Pb-eSOMA-02 (0.15 ± 0.02).

The tumor and organ doses absorbed for the different radioligands are indicated in Table 4. [^212^Pb]Pb-eSOMA-01 showed the highest absorbed dose to the tumor compared to the reference [^212^Pb]Pb-DOTAM-TATE and [^212^Pb]Pb-eSOMA-02. Furthermore, the lowest absorbed dose to the kidneys (the main limiting organ for PRRT of NETs) was also found for [^212^Pb]Pb-eSOMA-01, resulting in a more favorable kidneys/tumor absorbed dose ratio for [^212^Pb]Pb-eSOMA-01 compared to [^212^Pb]Pb-DOTAM-TATE. 

## 3. Discussion

Targeted alpha therapy is a promising therapeutic alternative to improve the outcome of radionuclide therapy for NETs. In this study, we focused on the development of SSTR2 radioligands that could be labeled with lead-212 and performed a head-to-head comparison to [^212^Pb]Pb-DOTAM-TATE, a reference compound that is currently under evaluation in phase II clinical trials [18]. Our radioligands were based on the SSTR2 agonist octreotate (TATE) and contained TCMC or DO3AM, two chelators known to enhance the stability of lead radionuclide-chelator complexes in vivo compared to other commonly used chelators, such as DOTA [19]. Two linkers (4-(aminomethyl)cyclohexane-1-carbonyl (Amcha) and 4-amino-1-carboxymethyl-piperidinyl (Pip)) were inserted between the chelator and the peptide sequence to preserve the binding of the ligand to SSTR2 and influence the pharmacokinetic properties. The Amcha linker was previously reported by dos Santos et al. in combination with the DO3AM chelator and a PSMA-targeting ligand to give CA012 [20]. [^203^Pb]Pb-CA012 demonstrated higher uptake in PSMA+ C4-2 tumor xenografts than [^203^Pb]Pb-CA011, the analog without linker. Amouroux et al. coupled the cationic linker Pip to a kallidin derivative to image the bradykinin B1 receptor with [^68^Ga]Ga-P04168 [21]. The Pip linker led to an improved biodistribution profile and better tumor visualization than the other linkers tested in this study. Therefore, both linkers were included in the design of our SSTR2 radioligands to determine if they would have a similar beneficial effect on the biodistribution of DOTAM-TATE.

Radiolabeling of the ligands with ^203/212^Pb was performed with a high radiochemical yield and purity at room temperature and at a pH maintained between 5 and 6. These radiolabeling conditions are well suited for the labeling of sensitive biomolecules [22]. We did not observe radiolytic degradation of the radiolabled compounds, except for eSOMA-03, despite the presence of quenchers (e.g., ascorbic acid and gentisic acid), which are preventing radiolysis in the final formulation [23]. Radiolysis is a form of decomposition occurring in the presence of radical species, such as hydroxyl radicals (•OH) or superoxide (•O2). We speculated that the instability of eSOMA-03 might originate from the hydrolysis of the thiourea bond [24,25,26,27]. Moreover, unlike eSOMA-03, all radiolabeled compounds were stable in mouse serum, demonstrating their inertness toward peptidase digestion. eSOMA-03 was therefore withheld from further investigations as it would fail during in vivo evaluation.

The LogD_7_._4_ values obtained for our SSTR2-targeting ligands were negative, but we noticed a decreased hydrophilicity compared to previous octreotate analogs, such as [^68^Ga]Ga-DOTA-TATE (LogD_7_._4_ = −3.69 ± 0.02) [28]. The lower hydrophilicity might be the result of the change of the chelator from DOTA containing three carboxylic acid groups to DO3AM bearing three amide groups. eSOMA-01 and eSOMA-02 showed a slightly lower LogD_7_._4_ value than DOTAM-TATE and eSOMA-03, which highlights the influence of the linker on the hydrophilicity of the radioligand. The three new ligands exhibited a binding affinity for SSTR2 that was slightly better than that of the gold standard, DOTA-TATE. However, their IC_50_ values were 3- to 4-fold higher than the IC_50_ value of DOTAM-TATE. This difference could be explained by the sensitivity of the binding of octreotate to SSTR2 when chemical modifications are performed at the *N*-terminus of the peptide [29,30]. The IC_50_ value of [^nat^Pb]Pb-DOTAM-TATE was comparable to the Kd value reported by Stallons and coworkers (7.6 nM vs. 12.9 nM, respectively) [18]. Complexation with lead had a slight effect on the binding affinity since all complexed compounds showed a 1.8–8.3-fold higher IC_50_ value compared to the non-complexed analogs. However, after Pb-complexation, the eSOMA derivatives exhibited lower IC_50_ values than [^nat^Pb]Pb-DOTAM-TATE (5.2, 6.8, and 5.6 nM for [^nat^Pb]Pb-eSOMA-01, -02, and -03, respectively, vs. 7.6 nM for [^nat^Pb]Pb-DOTAM-TATE), demonstrating that metal complexation had more influence on DOTAM-TATE than the three new ligands. The presence of the linkers in the eSOMA derivatives reduced the effect on binding to the receptor caused by the change of the secondary and tertiary structures of the peptides due to metal complexation. Furthermore, it has been previously reported that the complexation of SSTR2 ligands with different metals influences more or less the binding to the receptor [30,31]. Therefore, a good chelate-metal combination must be found.

eSOMA-01 and eSOMA-02 were first labeled with ^203^Pb, an imaging surrogate of ^212^Pb, and evaluated in a human small-cell lung cancer (NCI-H69) xenograft model overexpressing SSTR2. Tumor uptake at 1 h post-injection was ~4% ID/mL for both radioligands and slowly decreased to ~2–3% ID/mL at 24 h. Tworowska et al. reported an uptake of 13% ID/g at 1 h post-injection in AR42J tumors for [^203^Pb]Pb-DOTAM-TATE [32]. This tumor uptake is higher than the uptake observed with our compounds in H69 tumors. However, the difference could be explained by the fact that the AR42J cell line is known to have a 3-fold higher SSTR2 expression than the NCI-H69 cell line [33]. A high kidney uptake was also observed for [^203^Pb]Pb-eSOMA-01 and [^203^Pb]Pb-eSOMA-02. Kidney uptake was expected and is often reported in preclinical studies dealing with PRRT of NET due to the reabsorption of the peptide by the proximal tubules [34,35]. A similar uptake in kidneys (~13% ID/g) was reported by Tworowska and coworkers for [^203^Pb]Pb-DOTAM-TATE at 24 h [36].

Biodistribution of [^212^Pb]Pb-eSOMA-01 and [^212^Pb]Pb-eSOMA-02 showed a similar profile to the ^203^Pb-analogs, as expected due to the chemical similarities of ^203^Pb and ^212^Pb. Amongst the compounds evaluated, [^212^Pb]Pb-eSOMA-01 showed the highest tumor uptake, confirming the findings of dos Santos et al. on PSMA-targeting ligands containing an Amcha linker [20]. [^212^Pb]Pb-DOTAM-TATE showed a lower tumor uptake (~8–9% ID/g) than the uptake reported by Tworowska et al. (~20% ID/g). This difference could be explained by the varying levels of SSTR2 expression in the xenograft models. The kidney uptake of [^212^Pb]Pb-DOTAM-TATE (from 54 ± 5 at 1 h to 29 ± 6% ID/g at 24 h) was approximately 3-fold higher than the uptake reported by Tworowska et al. (from 21% ID/g at 1 h to 10% ID/g at 24 h). However, the pancreas uptake of [^212^Pb]Pb-DOTAM-TATE was lower at 1 h and 4 h (5.19 ± 0.84% ID/g and 1.14 ± 0.13% ID/g, respectively) than the values reported by Tworowska et al. (32% ID/g at 1 h and 15% ID/g at 4 h). The difference observed can be explained by the fact that two different mouse models were used (Balb/c nude mice in our study and CD-1 mice for Tworowska et al.) [37]. A therapeutic index corresponding to the ratio of the absorbed dose to the tumor and the absorbed dose to the main limiting organ (i.e., the kidneys) was defined to better evaluate the potential of the radioligands for therapy. In our study, [^212^Pb]Pb-eSOMA-01 presented a better therapeutic index than [^212^Pb]Pb-DOTAM-TATE and [^212^Pb]Pb-eSOMA-02 (1.6- and 3.0-fold lower, respectively). Thus, [^212^Pb]Pb-eSOMA-01 was identified as the best candidate for TAT of NETs among the SSTR2-targeting radioligands tested.

For therapy, the dose activity injected is usually determined by the maximum tolerated absorbed dose (MTD) of the dose-limiting organs, typically the kidneys for PRRT of NETs [38]. A MTD to the kidneys of 18–23 Gy was used by Tworowska et al. for [^212^Pb]Pb-DOTAM-TATE in mice [36,39]. Based on this limit, the maximum dose injected into mice would be around 0.2 MBq for [^212^Pb]Pb-eSOMA-01. However, the dose calculation assumed homogeneous uptake in the kidneys, whereas specific uptake in the tubuli might lead to lower absorbed doses in the glomeruli, especially with the short radiation range of the α particles [40]. Consequently, the dose absorbed by the kidneys could be less than the dose calculated. Autoradiography of the kidneys could provide more accurate information about the exact location of the radionuclide in this organ to refine the estimation of the absorbed dose. As a result, an absorbed dose to the tumor of 7 Gy was estimated for an injected activity of 0.2 MBq. However, a minimum dose of 20 Gy is typically needed in mice to achieve optimal therapeutic efficacy [41]. Therefore, an effort to reduce the dose absorbed by the kidneys is required in order to enhance the dose administered while keeping nephrotoxicity as low as possible. Co-injection of a basic amino acid cocktail or plasma expander (e.g., Gelofusine) could be explored to reduce renal retention of [^212^Pb]Pb-eSOMA-01 [42,43,44]. This strategy has already been used in preclinical and clinical studies with [^212^Pb]Pb-DOTAM-TATE and showed a reduction in kidney uptake by 3-fold without affecting tumor uptake [16,18].

## 4. Materials and Methods

### 4.1. General Information

All chemicals and solvents were obtained from commercial suppliers and used without further purification, unless specified. DO3AM acetic acid and 2-chlorotrityl chloride resin were purchased from Chematech (Dijon, France) and Advanced Chemtech (Louisville, KY, USA), respectively. Chinese hamster ovary-K1 (CHO-K1) membranes overexpressing human SSTR2 were purchased from Perkin Elmer (Waltham, MA, USA). [^111^In]InCl_3_ was ordered from Curium (Petten, The Netherlands). The peptide sequence was synthesized manually using standard solid-phase synthesis protocols. Mass analyses were carried out using a Thermo Fischer Scientific LC/MS system with electrospray ionization (ESI) (Breda, The Netherlands). Quality control and purification of the synthesized ligands were performed by high performance liquid chromatography (HPLC) using a Waters 2695 system (Etten-Leur, The Netherlands) equipped with a diode array detector 2998 and a radioactivity detector from Canberra (Zadik, Belgium). The HPLC and LC/MS systems were controlled by Empower3 and Xcalibur softwares, respectively. Instant thin-layer chromatography plates (iTLC) were analyzed by a bSCAN radio-chromatography scanner from Brightspec (Antwerp, Belgium) equipped with a sodium iodide detector. Activity measurements were performed using a VDC-405 dose calibrator from Comecer (Joure, The Netherlands). The radioactive samples used for the determination of LogD_7_._4_, in vitro assays, and in vivo uptake in tissues were counted using a Wizard 2480 gamma counter from Perkin Elmer (Waltham, MA, USA). It is noteworthy to mention that the activity of ^212^Pb samples was counted using a window energy of 204–267 keV.

### 4.2. HPLC Conditions

The analysis of the products was performed by HPLC on an analytical column (Phenomenex Gemini C_18_, 5 µm, 250.0 × 4.6 mm) at a flow rate of 1 mL/min and with a mobile phase consisting of: A (0.1% TFA in water (*v/v*)) and B (0.1% TFA in acetonitrile (*v/v*)). The elution of the products was performed according to the following gradient: 0–3 min: 5% B, 3–23 min: 5–100% B, and 23–27 min: 100% B.

Purification of the peptides was performed using a semi-preparative column (Phenomenex Luna RP-C_18_ column, 10 μm, 250 × 10 mm) at a flow rate of 3 mL/min according to: Condition 1: a gradient of acetonitrile (10% to 90%) in water containing 0.1% TFA over 20 min; Condition 2: an isocratic elution with 25% acetonitrile in water containing 0.1% TFA; or Condition 3: a gradient of acetonitrile (5% to 100%) in water containing 0.1% TFA over 20 min.

The analysis of the radioactive products was performed by radio-HPLC with: Method 1: Acquity UPLC HSS C_18_ column (1.8 µm, 50 × 2.1 mm); flow rate of 0.5 mL/min; mobile phase composition: A (0.1% TFA in water (*v*/*v*)) and B (0.1% TFA in acetonitrile (*v*/*v*)); gradient elution: 0–0.2 min: 5 to 25% B, 0.2–2.5 min: 25% B, 2.5–2.8 min: 25 to 100% B. Method 2: Nucleosil 120-5 C_18_ column (5 µm, 125.0 × 4.6 mm); flow rate of 1 mL/min; mobile phase consisting of: A (0.1% TFA in water (*v/v*)) and B (0.1% TFA in acetonitrile (*v/v*)); gradient elution: 0–2 min: 5 to 30% B, 2–10 min: 30% B, 10–12 min: 30 to 100%, and 12–14 min: 100% B. Fractions were collected (1 fraction/0.5 min) and measured in the gamma counter.

### 4.3. Synthetic Method

#### 4.3.1. d-Phe-cyclo[Cys-Tyr(tBu)-d-Trp(Boc)-Lys(Boc)-Thr(tBu)-Cys]-Thr(tBu)-resin (4)

The d-Phe-Cys(Acm)-Tyr(*t*Bu)-d-Trp(Boc)-Lys(Boc)-Thr(*t*Bu)-Cys(Acm)-Thr(*t*Bu)-resin was synthesized using a *N*^𝛼^-Fmoc solid-phase peptide synthesis (SPPS) strategy. The conjugation of Fmoc-protected amino acid (4.0 equiv.) to the 2-chlorotrityl chloride resin was carried out in dimethylformamide (DMF) using hexafluorophosphate azabenzotriazole tetramethyl uronium (HATU; 3.8 equiv.) and *N*,*N*-diisopropylethylamine (DIPEA; 7.8 equiv.) for 45 min. Fmoc deprotection was accomplished by treating the resin with a 20% solution of 4-methylpiperidine (4-MP) in DMF for 15 min. Amide formation and Fmoc deprotection were monitored by the TNBS test. Coupling or Fmoc deprotection were performed twice when the reaction was not completed. The peptide synthesis was started by loading Fmoc-l-Thr(*t*Bu)-OH onto the 2-chlorotrityl chloride resin (0.25 g, average loading capacity: 1.6 mmol/g). The resin was shaken for 90 min at room temperature (rt). The resin was capped using 8 mL of dichloromethane/methanol/DIPEA (*v*/*v*/*v* = 80:15:5) for 15 min at rt. Subsequent Fmoc deprotection and coupling with Fmoc-L-Cys(Acm)-OH, Fmoc-l-Thr(*t*Bu)-OH, Fmoc-l-Lys(Boc)-OH, Fmoc-d-Trp(Boc)-OH, Fmoc-l-Tyr(*t*Bu)-OH, Fmoc-l-Cys(Acm)-OH, and Fmoc-d-Phe-OH were achieved following the protocols described above. After the ultimate Fmoc deprotection, the protected linear peptide was cyclized on resin by using thallium(III) trifluoroacetate (Tl(TFA)_3_, 2.0 equiv.) in DMF for 1 h at room temperature. The cyclization was monitored by analytical HPLC.

#### 4.3.2. DO3AM-d-Phe-cyclo[Cys-Tyr-d-Trp-Lys-Thr-Cys]-Thr-OH (DOTAM-TATE)

A fraction of the cyclic peptidyl resin **4** (40 µmol) was swollen in DMF. DO3AM acetic acid (50 mg, 120 µmol, 3 equiv.), PyBop (65 mg, 120 µmol, 3.0 equiv.), and DIPEA (70 µL, 400 µmol, 10.0 equiv.) in 1 mL of DMF were added to the resin, and the mixture was stirred overnight at rt. Conjugation of the chelator was confirmed by analytical HPLC after cleavage and deprotection of a small sample of peptide. After completion of the reaction, the resin was washed with DMF (5 × 3 mL) and dichloromethane (3 × 3 mL). A solution of TFA/H_2_O/TIPS (*v*/*v*/*v* = 95:2.5:2.5) was added to the resin, and the mixture was stirred for 2 h at rt. The solution was removed from the resin by filtration, and ice-cold diethyl ether was added to the filtrate. The precipitate, collected by centrifugation, was purified by semi-preparative HPLC (Condition 2) to give 6.3 mg of DOTAM-TATE as a white solid (11%). Purity > 99%. ESI-MS: m/z calc’ for C_65_H_93_N_17_O_16_S_2_ 1431.64; found 1431.44 [M]^+^.

#### 4.3.3. DO3AM-Amcha-d-Phe-cyclo[Cys-Tyr-d-Trp-Lys-Thr-Cys]-Thr-OH (eSOMA-01)

4-(Fmoc-aminomethyl)cyclohexanecarboxylic acid (Fmoc-Amcha-OH; 61 mg, 160 µmol, 4 equiv.) was dissolved in a solution of 0.38 M HATU (400 µL, 152 µmol, 3.8 equiv.). After complete dissolution, a solution of 0.78 M DIPEA (400 µL, 312 µmol, 7.8 equiv.) was added, and the mixture was directly added to the resin 4 (40 µmol). The mixture was stirred for 60 min at rt. The coupling was monitored by the TNBS test. After the Fmoc deprotection, the coupling of DO3AM and the global deprotection were performed following the protocol described for DOTAM-TATE. The peptide was purified by semi-preparative HPLC (Condition 1) to give 7.4 mg of eSOMA-01 as a white solid (12%). Purity > 99%. ESI-MS: m/z calc’ for C_73_H_106_N_18_O_17_S_2_ 1570.74; found 1571.71 [M+H]^+^.

#### 4.3.4. DO3AM-Pip-d-Phe-cyclo[Cys-Tyr-d-Trp-Lys-Thr-Cys]-Thr-OH (eSOMA-02)

The coupling of Fmoc-4-amino-1-piperidineacetic acid (Fmoc-Pip-OH), DO3AM, and the global deprotection were performed following the protocol described for eSOMA-01. The peptide was purified by semi-preparative HPLC (Condition 1) to give 4.2 mg of eSOMA-02 as a white solid (7%). Purity > 98%; ESI-MS: m/z calc’ for C_72_H_105_N_19_O_17_S_2_ 1571.74; found 1572.62 [M+H]^+^.

#### 4.3.5. d-Phe-cyclo[Cys-Tyr-d-Trp-Lys(ivDde)-Thr-Cys]-Thr (5)

d-Phe-Cys(Acm)-Tyr(*t*Bu)-d-Trp(Boc)-Lys(ivDde)-Thr(*t*Bu)-Cys(Acm)-Thr(*t*Bu)-resin was synthesized by a SPPS strategy similar to the synthesis of 4. Fmoc deprotection and coupling with Fmoc-l-Thr(*t*Bu)-OH, Fmoc-l-Cys(Acm)-OH, Fmoc-l-Thr(*t*Bu)-OH, Fmoc-l-Lys(ivDde)-OH, Fmoc-D-Trp(Boc)-OH, Fmoc-l-Tyr(*t*Bu)-OH, Fmoc-l-Cys(Acm)-OH, and Fmoc-d-Phe-OH were achieved following the same protocols described above. After the final Fmoc deprotection, the protected linear peptide was cyclized on resin by using Tl(TFA)_3_ (2.0 equiv.) in DMF for 1 h at rt. The cyclization was monitored by analytical HPLC. After completion of the reaction, the resin was washed with dichloromethane (3 × 3 mL). A solution of TFA/H_2_O/TIPS (*v*/*v*/*v* = 95:2.5:2.5) was added to the resin, and the mixture was stirred for 2 h at rt. The solution was removed from the resin by filtration, and ice-cold diethyl ether was added to the filtrate. The precipitated peptide was collected by centrifugation and purified by semi-preparative HPLC (Condition 3) to give 110 mg of peptide 5 as a white solid (22%). ESI-MS: m/z calc’ for C_62_H_82_N_10_O_14_S_2_ 1254.55; found 1255.54 [M+H]^+^.

#### 4.3.6. TCMC-Bn-thiourea-(d-Phe-cyclo[Cys-Tyr-d-Trp-Lys(ivDde)-Thr-Cys]-Thr-OH (6)

*p*-SCN-Bn-TCMC (50 mg, 72 µmol, 2 equiv.), PyBop (37 mg, 72 µmol, 3.0 equiv.) and DIPEA (63 µL, 360 µmoL, 10.0 equiv.) in 1 mL of DMF were added to the peptide 5 (45 mg, 36 µmol, 1 equiv.). The mixture was stirred overnight at rt. DMF was removed under a high vacuum, and the crude product was purified by semi-preparative HPLC (Condition 3) to give 11 mg of the peptide 6 as a white solid (17%). ESI-MS: *m*/*z* calc’ for C_86_H_119_N_19_O_18_S_3_ 1801.83; found 1802.76 [M + H]^+^.

#### 4.3.7. TCMC-Bn-thiourea-(d-Phe-cyclo[Cys-Tyr-d-Trp-Lys-Thr-Cys]-Thr-OH (eSOMA-03)

6 (11 mg, 6,1 µmol, 1 equiv.) was dissolved in a solution of 2% hydrazine in DMF (2 mL) and the mixture was stirred for 1 h at rt. DMF was removed under high vacuum, and the product was purified by semi-preparative HPLC (Condition 3) to give 2 mg of the peptide eSOMA-03 as a white solid (17%). Purity > 95%; ESI-MS: *m*/*z* calc’ for C_73_H_101_N_19_O_16_S_3_ 1595.68; found 1595.95 [M]^+^.

### 4.4. Complexation with the Lead ICP Standard

Pb(NO_3_)_2_ (5 equiv., 0.1 g/L) in 2–3% HNO_3_ was added to a mixture of DOTAM-TATE or eSOMA-(01-03) (10^−4^ M), sodium acetate (10 μL, 2.5 M), and H_2_O (final volume of 250 μL). The mixture was incubated for 20 min at rt. The reaction was monitored by analytical HPLC. Diethylenetriaminepentaacetic acid (DTPA; 5 μL, 3 mg/mL) was added to complex the remaining free lead. The solution was used without further purification.

### 4.5. Radiolabeling with ^203^Pb

Before labeling, the concentration of all the peptides was determined via titration according to a method previously described [45]. [^203^Pb]PbCl_2_ (40 MBq) in 0.5 M HCl (Lantheus Medical Imaging; North Billerica, MA, USA) was added to a mixture of DOTAM-TATE or eSOMA-(01-03) (1 nmol), ascorbic acid/gentisic acid (10 μL, 50 mM), sodium acetate (30 μL, 2.5 M), and H_2_O (final volume of 140 μL). The mixture was incubated for 20 min at rt. The reaction was monitored by instant thin-layer chromatography (iTLC) on silica gel-impregnated glass fiber plates iTLC-SG (Agilent; Amsterdam, the Netherlands) eluted with a solution of sodium citrate (0.1 M, pH 5.0). DTPA (5 μL, 3 mg/mL) was added to complex the remaining free lead-203. An aliquot was taken and injected into radio-HPLC using Method 1.

### 4.6. Radiolabeling with ^212^Pb

[^212^Pb]PbCl_2_ (100 kBq) in 0.1 M HCl (NRG; Petten, the Netherlands) was added to a mixture of DOTAM-TATE or eSOMA-(01-03) (1 nmol), ascorbic acid/gentisic acid (10 μL, 50 mM), sodium acetate (30 μL, 2.5 M), and H_2_O (final volume of 140 μL). The mixture was incubated for 20 min at rt. The reaction was monitored by instant thin-layer chromatography on iTLC-SG sheets eluted with a solution of sodium citrate (0.1 M, pH 5.0), which were cut into 10 equal pieces and measured in a gamma counter. DTPA (5 μL, 3 mg/mL) was added to complex the remaining free lead-212. An aliquot was taken and injected into radio-HPLC using Method 2.

### 4.7. In Vitro Stability

The ^203^Pb-labeled compounds (~ 2 MBq) or the ^212^Pb-labeled compounds (~ 70 kBq) were incubated in 300 µL of phosphate buffered saline (PBS; 0.1 M, pH 7.4) or mouse serum at 37 °C. The stability of the radiolabeled peptides was monitored at 1, 4, and 24 h post-incubation. The samples in PBS were directly analyzed by radio-HPLC without any pretreatment. However, samples of mouse serum were mixed with an equal volume of acetonitrile to precipitate the proteins. The solution was centrifuged twice for 5 min at 13,000 rpm, and the supernatant was analyzed by radio-HPLC using Method 1 for lead-203 and Method 2 for lead-212.

### 4.8. LogD_7_._4_

Distribution coefficients (LogD_7_._4_) were determined by the shake-flask method. A sample containing the ^203^Pb-labeled compound (~1 MBq) was added to a vial containing PBS (600 µL, pH 7.4) and n-octanol (600 µL). The vial was vortexed vigorously and then centrifuged at 10,000 rpm for 10 min for phase separation. Samples (100 µL) of the n-octanol and aqueous phases were taken and measured by a gamma counter. The LogD_7_._4_ value was calculated by using the equation: LogD_7_._4_ = log [(counts in the n-octanol phase)/(counts in the aqueous phase)]. Measurements were performed in triplicate.

### 4.9. Binding Affinity

The binding affinity of DOTAM-TATE, eSOMA-01, eSOMA-02, eSOMA-03, [^nat^Pb]Pb-DOTAM-TATE, [^nat^Pb]Pb-eSOMA-01, [^nat^Pb]Pb-eSOMA-02, and [^nat^Pb]Pb-eSOMA-03 for SSTR2 was determined using a cell membrane-based competitive binding assay. Purified Chinese hamster ovary-K1 (CHO-K1) membranes overexpressing human SSTR2 were incubated with [^111^In]In-DOTA-TATE and a competing non-radioactive ligand in a 96-well, 1.2 μm glass fiber filter plate (EMD Millipore; Darmstadt, Germany). Prior to the assay, the plate filters were pre-soaked in 0.1% polyethyleneimine for 1 h at ambient temperature. Membranes (25 μg/well, from a stock of 1.5 µg/uL or 400 U), [^111^In]In-DOTA-TATE (0.05 nM), and various concentrations of competing peptides (10 μM to 1 pM) diluted in assay buffer (25 mM HEPES, pH 7.4, 10 mM MgCl_2_, 1 mM CaCl_2_, 0.5% BSA) were incubated for 1 h at 27 °C with moderate shaking. Once complete, the medium was aspirated through the filters, followed by six washes with 200 μL of ice-cold buffer (50 mM Tris-HCl, pH 7.4, 0.2% BSA). Each filter was removed and measured with a gamma counter. DOTA-TATE was used as a positive control for affinity determination. The 50% inhibition constant (IC_50_) was calculated by fitting the data to a one-site Fit-Ki curve in GraphPad Prism v9.

### 4.10. Cell Culture

NCI-H69 cells (ATCC, Manassas, VA, USA) were cultured in Rosewell Park Medium Institute 1638 medium (RPMI-1638) (Sigma-Aldrich; Darmstadt, Germany) supplemented with penicillin (50 units/mL), streptomycin (50 μg/mL), and 10% fetal calf serum (FCS). Cells were cultured at 37 °C and 5% CO_2_.

### 4.11. Animal Models

Six-week-old male Balb/c nu/nu-specific and opportunistic pathogen-tree (SOPF) mice (Janvier Labs, Le Genest-Saint-Isle, France) were housed in individually ventilated cages, with four mice per cage. Upon arrival, the mice were acclimated for 1 week with access to food and water ad libitum. Mice were subcutaneously inoculated on the right shoulder with NCI-H69 cells (5 × 10^6^ cells suspended in 100 μL of 1/3 Matrigel (Corning Inc.; Corning, NY, USA) and 2/3 Hank’s balanced salt solution (Gibco; Paisley, UK). NCI-H69 xenografts were allowed to grow for 3 weeks. Tumor sizes were 391 ± 173 mm^3^ at the start of the studies.

### 4.12. In Vivo Single Photon Emission Computed Topology/Computer Tomography (SPECT/CT)

Mice (*n* = 4 per compound) were intravenously injected in the tail vein with 100 μL of [^203^Pb]Pb-eSOMA-01 (12.31 ± 1.44 MBq, 0.5 nmol) or [^203^Pb]Pb-eSOMA-02 (13.06 ± 1.44 MBq, 0.5 nmol) containing Kolliphor HS 15 (Merck; Haarlerbergweg, The Netherlands) in PBS (0.06 mg/mL). At 1, 4, and 24 h post-injection (p.i.), mice were imaged in a prone position on a heated bed under 2% isoflurane/O_2_ anesthesia in a dedicated small-animal PET/SPECT/CT VECTOR5 scanner (MILabs B.V.; Utrecht, The Netherlands) with a high-sensitivity pinhole collimator (HE-GP-M, 1.6 mm pinholes). Whole-body SPECT images were acquired over one frame of 20 min (for 1 and 4 h time points) and two frames of 20 min (for 24 h time points) using a spiral scan in normal scan mode, in list-mode acquisition. This was followed by a whole-body normal CT scan (angle step 0.75 degrees, 50 kV tube voltage, 0.21 mA tube current). Reconstruction of the SPECT images was performed using the similarity-regulated SROSEM method (MILabs Rec 11.00 software), performing five iterations with a voxel size of 0.4 mm, using 72 keV ± 30% and 280 keV ± 8% energy windows for lead-203. Reconstructed volumes of SPECT scans were post-filtered with an isotropic three-dimensional Gaussian filter of 1 mm full width at half-maximum. Image processing and analyses of the reconstructed data were performed using the PMOD image analysis software version 3.9 (PMOD Technologies; Zurich, Switzerland) to calculate the percentage of injected dose per mL (% ID/mL). An Eppendorf tube filled with a solution of lead-203 of known activity was measured to determine the calibration factor.

### 4.13. Ex-Vivo Biodistribution with Lead-203

To determine the biodistribution of the compounds after imaging (1, 4, and 24 h p.i.), blood was collected via retro-orbital puncture under isoflurane/O_2_ anesthesia, after which the mice were sacrificed via cervical dislocation. The tumor and organs of interest (heart, lungs, liver, spleen, stomach, large intestine, small intestine, pancreas, kidneys, muscle, skin, and bone) were excised, washed in PBS, blotted dry, and collected into tubes. The blood, tumor, and relevant organs were weighed and measured with a gamma counter. The percentage of injected dose per gram (% ID/g) was determined for each tissue sample and corrected for both the injected volume and the % ID present at the injection site (the tail).

### 4.14. Biodistribution Study with Lead-212

Biodistribution studies were performed to determine tumor and organ uptake of [^212^Pb]Pb-DOTAM-TATE, [^212^Pb]Pb-eSOMA-01, and [^212^Pb]Pb-eSOMA-02. Animals (*n* = 4 for each time point) were injected intravenously (i.v.) with an average of 100 kBq/0.5 nmol. At 3 selected time points (1, 4, and 24 h) p.i., blood was collected via retro-orbital puncture under isoflurane/O_2_ anesthesia, after which the mice were sacrificed via cervical dislocation. The tumor and organs of interest (heart, lungs, liver, spleen, stomach, large intestine, small intestine, pancreas, kidneys, muscle, skin, and bone) were excised, washed in PBS, and blotted dry. To confirm receptor specificity of radioligand uptake, mice (*n* = 3) were co-injected with an excess (25 nmol) of the respective unlabeled peptide, and uptake in the organs of interest was determined at 24 h p.i. To determine the total injected radioactivity per animal, a calibration curve with lead-212 was established using the gamma counter. The percentage of injected dose per gram (% ID/g) was determined for each tissue sample and corrected for both the injected volume and the % ID present at the injection site (the tail).

### 4.15. Statistical Analysis

Statistical analysis and nonlinear regression were performed using GraphPad Prism 5 (GraphPad software, San Diego, CA, USA), and a Grubbs’ test (α = 0.05) was used to compare medians between groups. The Akaike information criterion was used to decide if a residual plateau needed to be included in the exponential fit. Data were reported as mean ± SEM (standard error of mean) for at least three independent replicates. The data sets were analyzed for significance using a one-way ANOVA using SigmaPlot 15.0 software. A *p*-value lower than 0.05 was considered statistically significant.

### 4.16. Dosimetry

Single exponential curves were fitted through the organ biodistribution time-activity data using Graphpad Prism. The number of disintegrations per injected activity per gram of tissue ([TIAC]) was calculated by integrating the single exponential curve folded with the ^212^Pb decay function with a half-life of 10.64 h. The TIAC per gram of tissue by the progenies of ^212^Pb: ^212^Bi, ^212^Po (64.06%), and ^208^Tl (35.94%) were assumed to be equal to ^212^Pb, as they are in equilibrium. The absorbed doses to the organs were calculated by using the ^212^Pb and its progenies’ S-values for the 25 g RADAR mouse phantom [46]. The absorbed doses were calculated with the MIRD equation $D=\underset{s}{\sum }m_{s}\times \left[TIAC_{s}\right]\times S\left(t\leftarrow s\right)$, with m_s_ being the source organ mass. A relative biological effect (RBE) of 5 was assumed for the alpha-radiation [47]. The uncertainty propagation in the dosimetry result was calculated [48].

## 5. Conclusions

In this study, we successfully developed and evaluated in preclinical models three new SSTR2-targeting ligands labeled with ^203/212^Pb for imaging and treatment of NETs. We demonstrated that eSOMA-01 is a promising theragnostic candidate. eSOMA-01 showed a superior biodistribution profile and therapeutic index than the actual reference, DOTAM-TATE. However, further investigations are required to reduce the absorbed dose to the kidneys, the dose-limiting organ for radionuclide therapy of NETs. Therapy studies will be conducted to compare the efficacy of [^212^Pb]Pb-eSOMA-01 with the clinical reference [^212^Pb]Pb-DOTAM-TATE.

## 6. Patents

D.C. and Y.S. have a pending patent application for the aforementioned compounds.

## Figures and Tables

**Table 1:** LogD_7_._4_ and stability in PBS and mouse serum of ^203^Pb-labeled DOTAM-TATE, eSOMA-01, eSOMA-02, and eSOMA-03.

Compound	LogD_7_._4_ ^b^	Stability in PBS(%) ^a^	Stability in PBS(%) ^a^
		1 h 4 h 24 h	1 h 4 h 24 h
[^203^Pb]Pb-DOTAM-TATE	−1.41 ± 0.01	99 99 99	99 99 99
[^203^Pb]Pb-eSOMA-01	−1.99 ± 0.03	100 100 100	100 100 100
[^203^Pb]Pb-eSOMA-02	−1.88 ± 0.03	97 97 97	97 97 97
[^203^Pb]Pb-eSOMA-03	−1.36 ± 0.06	70 40 15	85 70 60

^a^ Results are expressed as a percentage of intact labeled ligand after incubation at 37 °C (*n* = 1); ^b^ *n* = 3.

**Table 2:** Stability in PBS and mouse serum of ^212^Pb-labeled DOTAM-TATE, eSOMA-01, and eSOMA-02.

Compound	Stability in PBS(%) ^a^	Stability in PBS(%) ^a^
	1 h 4 h 24 h	1 h 4 h 24 h
[^212^Pb]Pb-DOTAM-TATE	99 98 95	99 99 97
[^212^Pb]Pb-eSOMA-01	99 97 94	96 96 93
[^212^Pb]Pb-eSOMA-02	97 95 95	99 99 96

^a^ Results are expressed as a percentage of intact labeled ligand after incubation at 37 °C (*n* = 1).

**Table 3:** Determination of the IC_50_ values by a competitive binding assay.

Compound	IC_50_ (nM)
DOTA-TATE	3.70 ± 0.18 ^a^
DOTAM-TATE	0.91 ± 0.18 ^a^
eSOMA-01	2.54 ± 0.20 ^a^
eSOMA-02	2.53 ± 0.19 ^a^
eSOMA-03	3.11 ± 0.05 ^a^
[^nat^Pb]Pb-DOTAM-TATE	7.60 ^b^
[^nat^Pb]Pb-eSOMA-01	5.29 ^b^
[^nat^Pb]Pb-eSOMA-02	6.83 ^b^
[^nat^Pb]Pb-eSOMA-03	5.64 ^b^

^a^ *n* = 2; ^b^ *n* = 1.

**Table 4:** Absorbed dose per administered activity (Gy/MBq; with RBE = 5) in H69-tumor xenograft and organs for [^212^Pb]Pb-DOTAM-TATE, [^212^Pb]Pb-eSOMA-01, and [^212^Pb]Pb-eSOMA-02.

Tissue	[^212^Pb]Pb-DOTAM-TATE	[^212^Pb]Pb-eSOMA-01	[^212^Pb]Pb-eSOMA-02
Tumor	26.61 ± 19.27	35.49 ± 9.89	14.73 ± 5.73
Lungs	2.06 ± 0.29	3.20 ± 0.40	2.55 ± 0.28
Liver	6.93 ± 0.64	9.03 ± 2.24	8.46 ± 1.96
Spleen	0.56 ± 0.14	0.73 ± 0.19	1.17 ± 0.49
Stomach wall	4.42 ± 1.30	5.78 ± 0.77	2.92 ± 0.33
Large Intestine	1.64 ± 0.30	2.03 ± 0.26	1.74 ± 0.39
Pancreas	3.65 ± 0.28	5.40 ± 0.14	4.36 ± 0.28
Kidneys	140.03 ± 17.95	121.73 ± 14.57	147.44 ± 36.20
K/T	5.26 ± 3.90	3.43 ± 1.10	10.01 ± 4.60

## Data Availability

Data is contained within the article and Supplementary Material.

## Supplementary Materials

The following supporting information can be downloaded at: https://www.mdpi.com/article/10.3390/ph16070985/s1. The following supporting information can be downloaded: Figure S1: HPLC chromatograms of DOTAM-TATE (A), eSOMA-01 (B), eSOMA-02 (C), and eSOMA-03 (D); Figure S2: Mass spectrometry of DOTAM-TATE (A), eSOMA-01 (B), eSOMA-02 (C), and eSOMA-03 (D);Figure S3: IC_50_ curves of the in vitro competitive binding assay for DOTAM-TATE, eSOMA-01, eSOMA-02, and eSOMA-03; Figure S4: IC50 curves of the in vitro competitive binding assay for [^nat^Pb]Pb-DOTAM-TATE, [^nat^Pb]Pb-eSOMA-01, [^nat^Pb]Pb-eSOMA-02, and [^nat^Pb]Pb-eSOMA-03; Figure S5: iTLC spectra of [^203^Pb]Pb-DOTAM-TATE (A), [^203^Pb]Pb-eSOMA-01 (B), [^203^Pb]Pb-eSOMA-02 (C), and [^203^Pb]Pb-eSOMA-03 (D); Figure S6: Radio-HPLC chromatograms of [^203^Pb]Pb-DOTAM-TATE (A), [^203^Pb]Pb-eSOMA-01 (B), [^203^Pb]Pb-eSOMA-02 (C), and [^203^Pb]Pb-eSOMA-03 (D). Figure S7: iTLC spectra of [^212^Pb]Pb-DOTAM-TATE (A), [^212^Pb]Pb-eSOMA-01 (B), [^212^Pb]Pb-eSOMA-02 (C), and [^212^Pb]Pb-eSOMA-03 (D); Figure S8: Radio-HPLC chromatograms of [212Pb]Pb-DOTAM-TATE (A), [^212^Pb]Pb-eSOMA-01 (B), [^212^Pb]Pb-eSOMA-02 (C), and [^212^Pb]Pb-eSOMA-03 (D). Table S1: Ex vivo biodistribution analysis of [^203^Pb]Pb-eSOMA-01 and [^203^Pb]Pb-eSOMA-02 at 24 h post-injection; Table S2: Ex vivo biodistribution analysis of [^212^Pb]Pb-DOTAM-TATE at 1, 4, and 24 h post-injection; Table S3: Ex vivo biodistribution analysis of [^212^Pb]Pb eSOMA-01 at 1, 4, and 24 h post-injection; Table S4: Ex vivo biodistribution analysis of [^212^Pb]Pb eSOMA-02 at 1, 4, and 24 h post-injection.
