# Supplementary material for: [212Pb]Pb-eSOMA-01: A Promising Radioligand for Targeted Alpha Therapy of Neuroendocrine Tumors
Source: Pharmaceuticals (Basel). 2023 Jul 10;16(7):985. doi: 10.3390/ph16070985 (PMC10384862; doi:10.3390/ph16070985)
Supplement: Supplementary file 1 [file pharmaceuticals-16-00985-s001.zip › pharmaceuticals-2470641-supplementary.pdf]

Supplemental information

[^{212}Pb]Pb-eSOMA-01: a Promising Radioligand for Targeted Alpha Therapy of Neuroendocrine Tumors.

Dylan Chapeau[1,2], Sofia Koustoulidou[1,2], Maryana Handula[1,2], Savanne Beekman[1,2], Corrina de Ridder[1,2], Debra Stuurman[1,2], Erik de Blois[1,2], Yulia Buchatskaya[3], Karlijn van der Schilden[3], Marion de Jong[1,2,†], Mark W. Konijnenberg[1,2], Yann Seimbille[1,2,4,*]

[1] Erasmus MC, University Medical Center Rotterdam, Department of Radiology and Nuclear Medicine, Rotterdam, The Netherlands

[2] Erasmus MC Cancer Institute, Rotterdam, The Netherlands

[3] Nuclear Research & Consultancy Group, Petten, The Netherlands

[4] TRIUMF, Life Sciences Division, Vancouver, Canada

† The author passed away

*Correspondence: y.seimbille@erasmusmc.nl ; Tel: +31 10-703-8961

Supplemental information

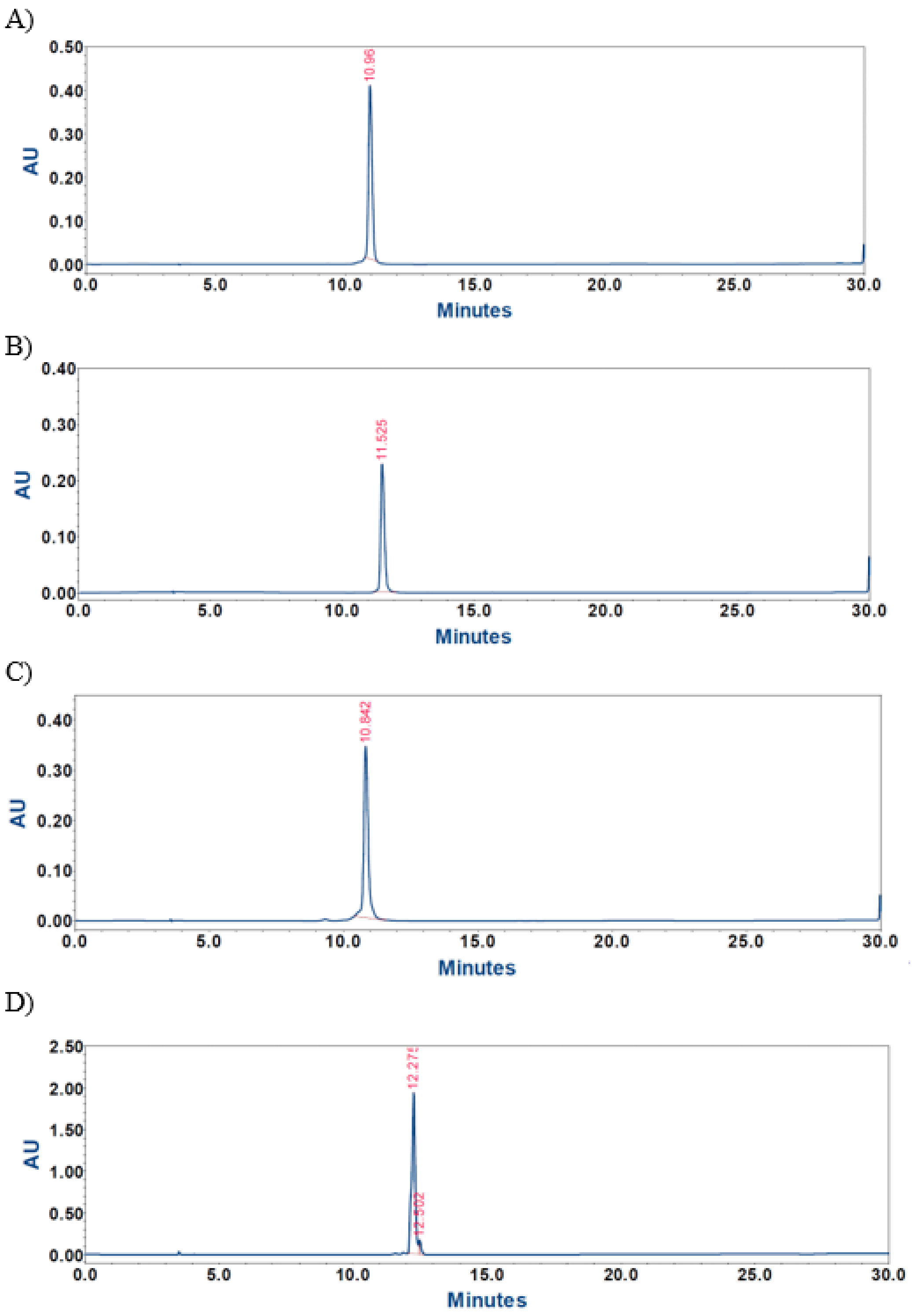

Figure S1*:* HPLC chromatograms of DOTAM-TATE (**A**), eSOMA-01 (**B**), eSOMA-02 (**C**) and eSOMA-03 **(D)**

A)

Supplemental information

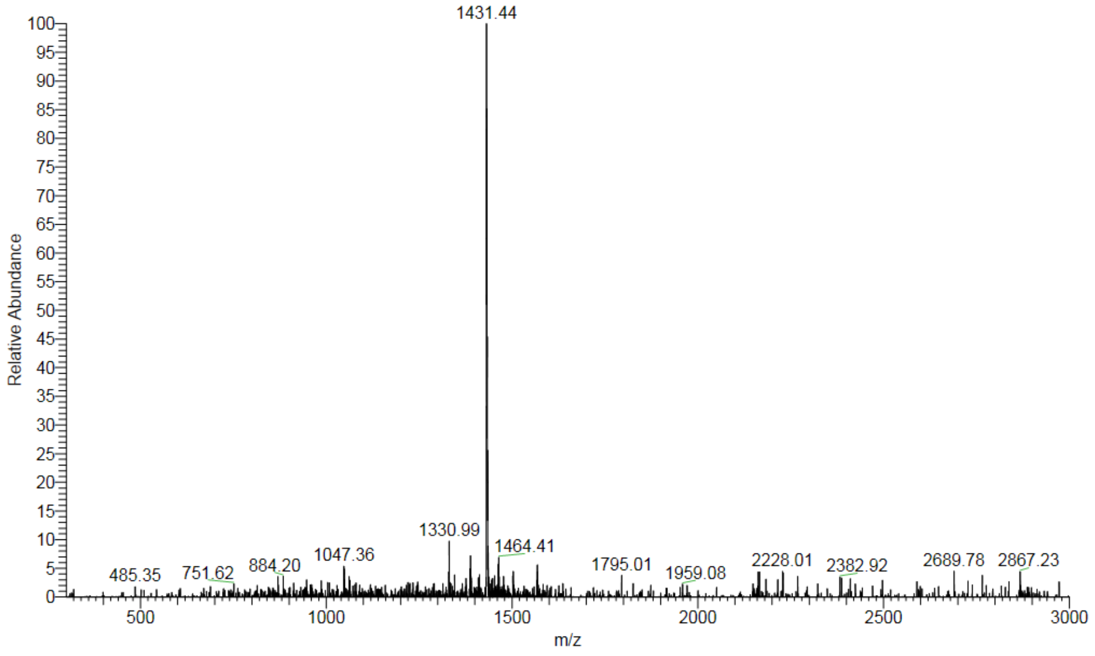

B)

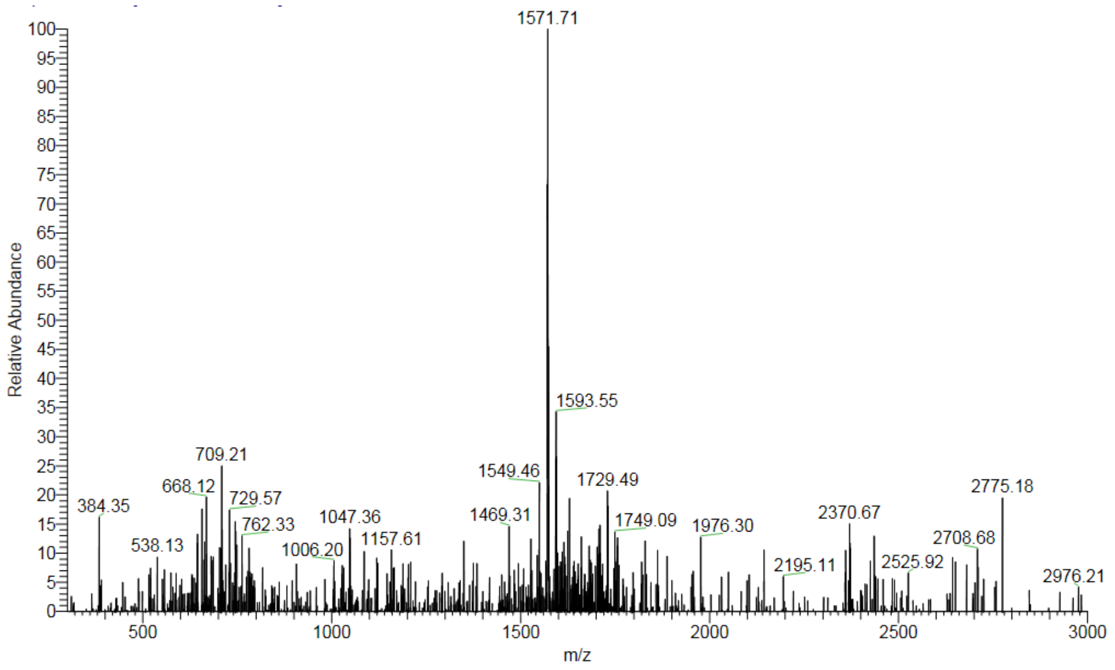

C)

Supplemental information

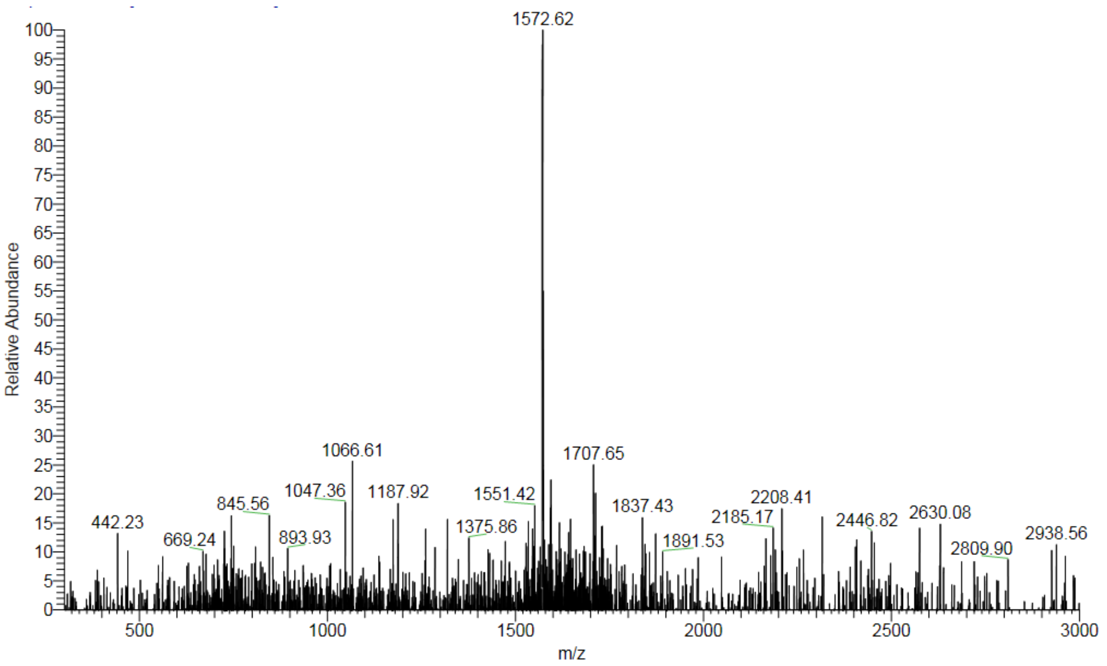

D)

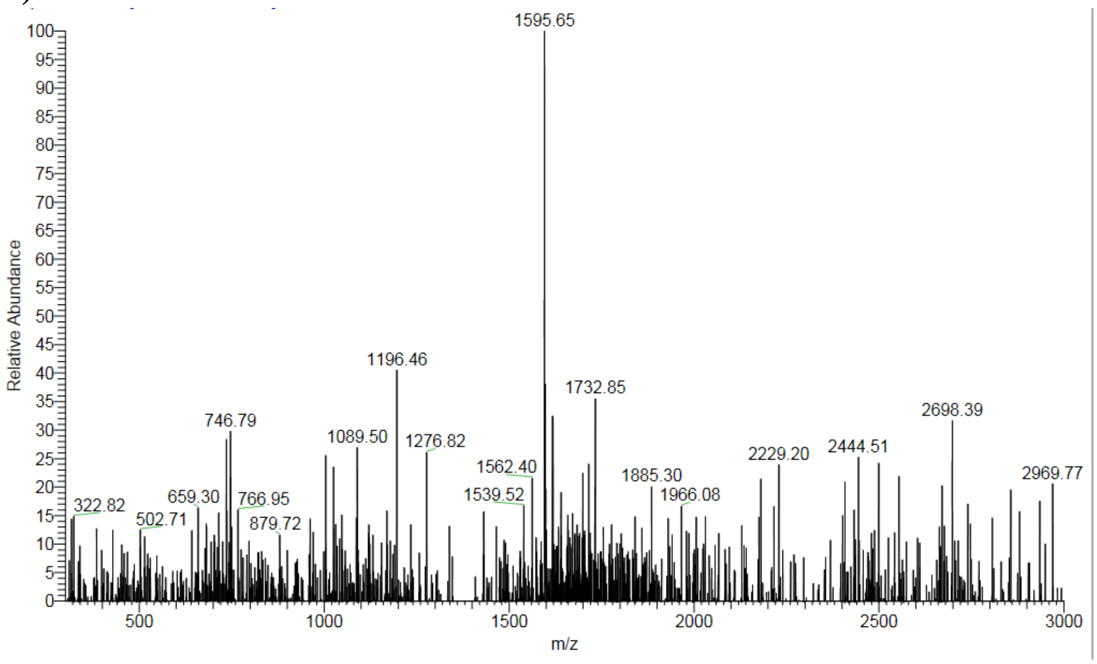

Figure S2: Mass spectrometry of DOTAM-TATE (**A**), eSOMA-01 (**B**), eSOMA-02 (**C**) and eSOMA-03 (**D**).

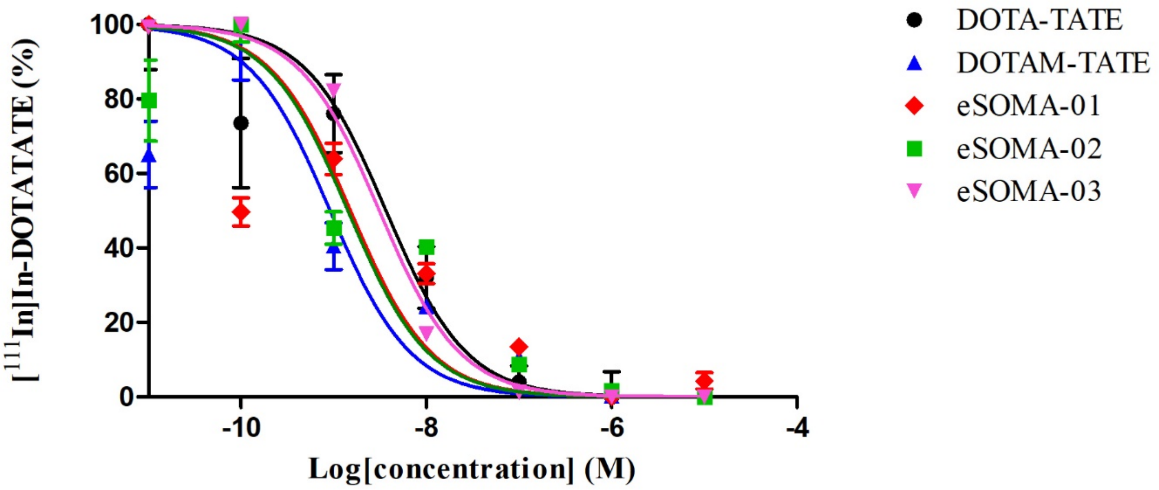

Figure S3: IC_{50} curves of the in vitro competitive binding assay for DOTAM-TATE, eSOMA-01, eSOMA-02 and eSOMA-03.

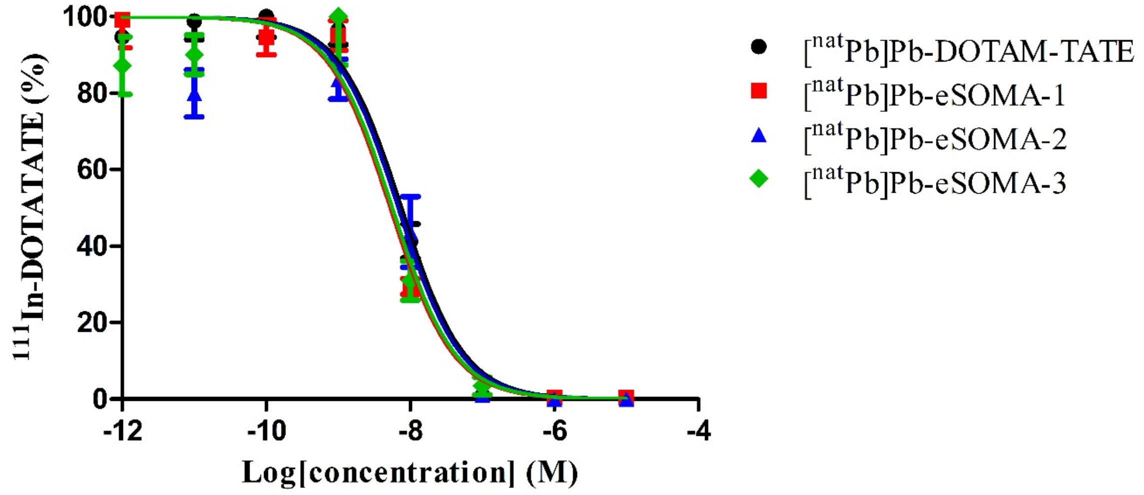

Figure S4: IC_{50} curves of the in vitro competitive binding assay for $[^{nat}Pb]$Pb-DOTAM-TATE, $[^{nat}Pb]$Pb-eSOMA-01, $[^{nat}Pb]$Pb-eSOMA-02 and $[^{nat}Pb]$Pb-eSOMA-03.

Supplemental information

A)

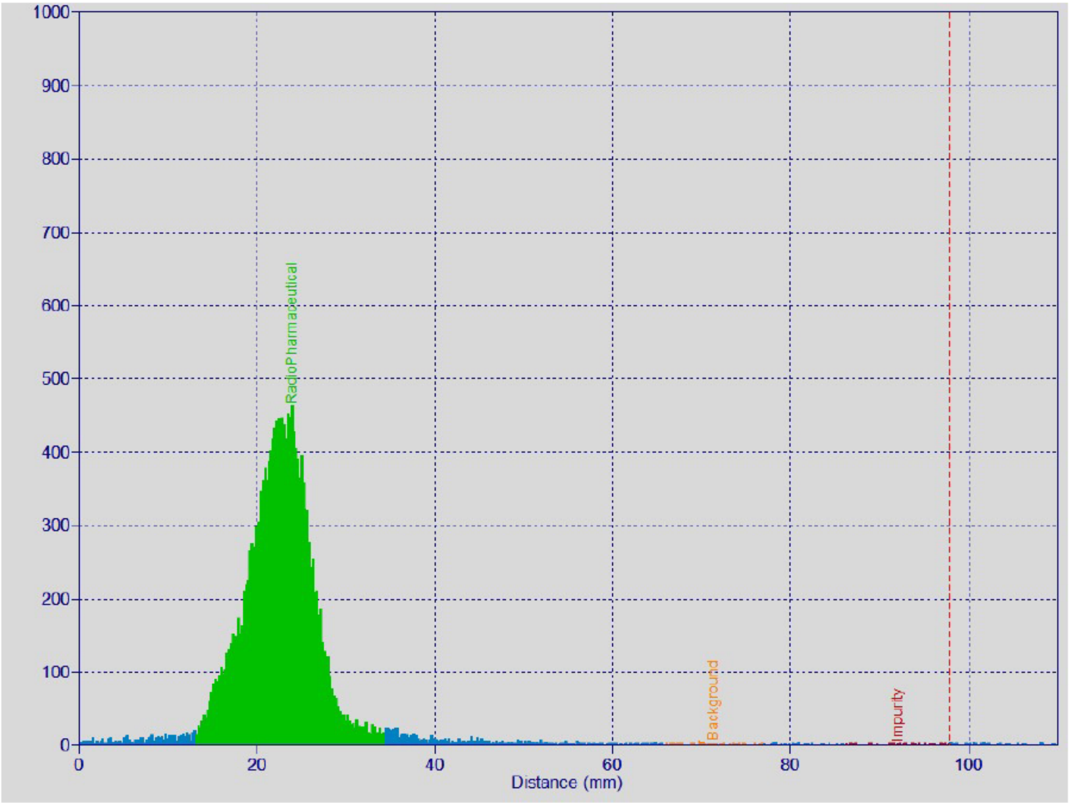

B)

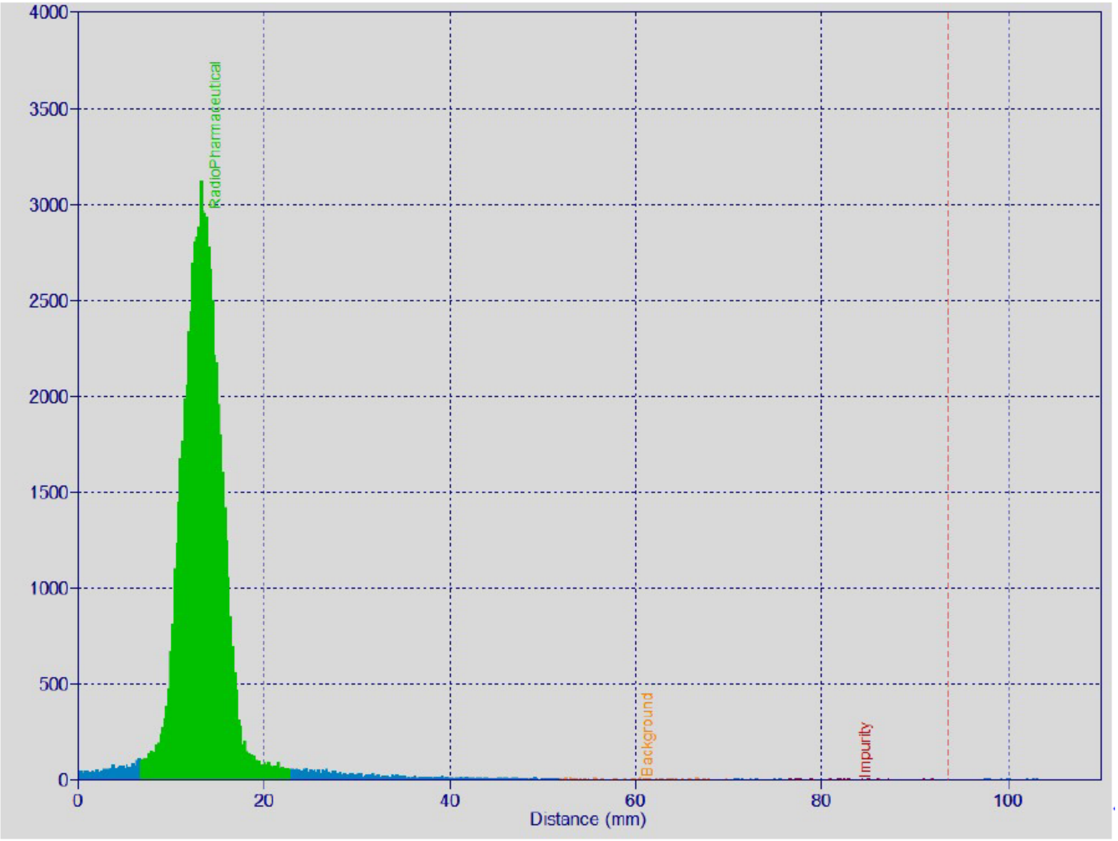

C)

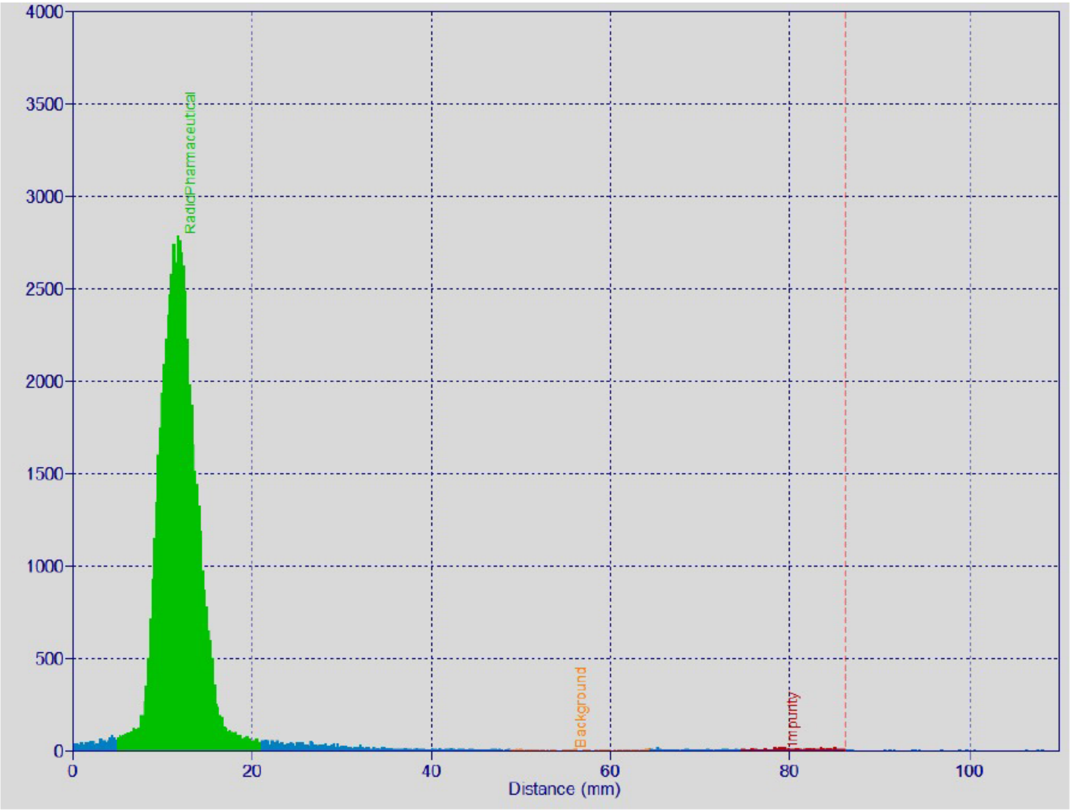

D)

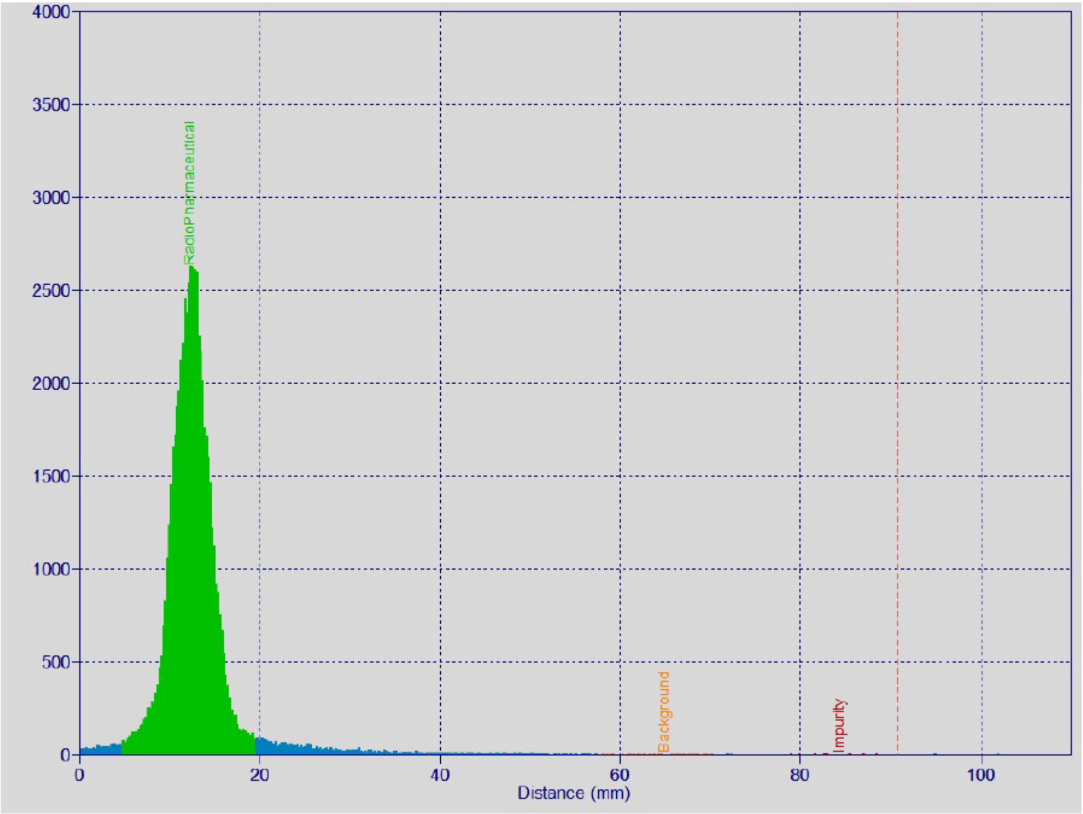

Figure S5: iTLC spectra of [^{203}Pb]Pb-DOTAM-TATE (**A**), [^{203}Pb]Pb-eSOMA-01 (**B**), [^{203}Pb]Pb-eSOMA-02 (**C**) and [^{203}Pb]Pb-eSOMA-03 (**D**).

Supplemental information

A)

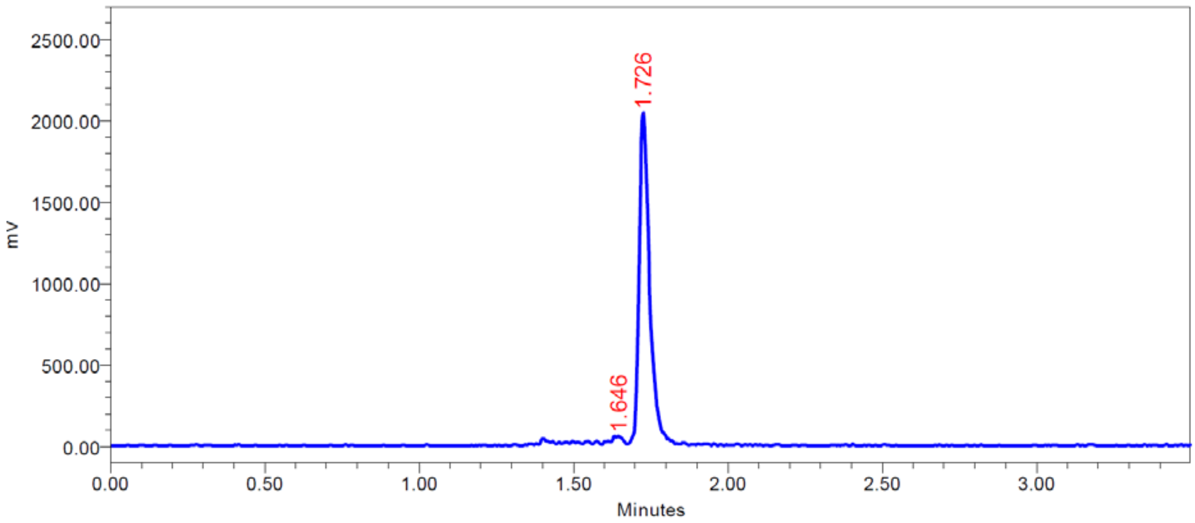

B)

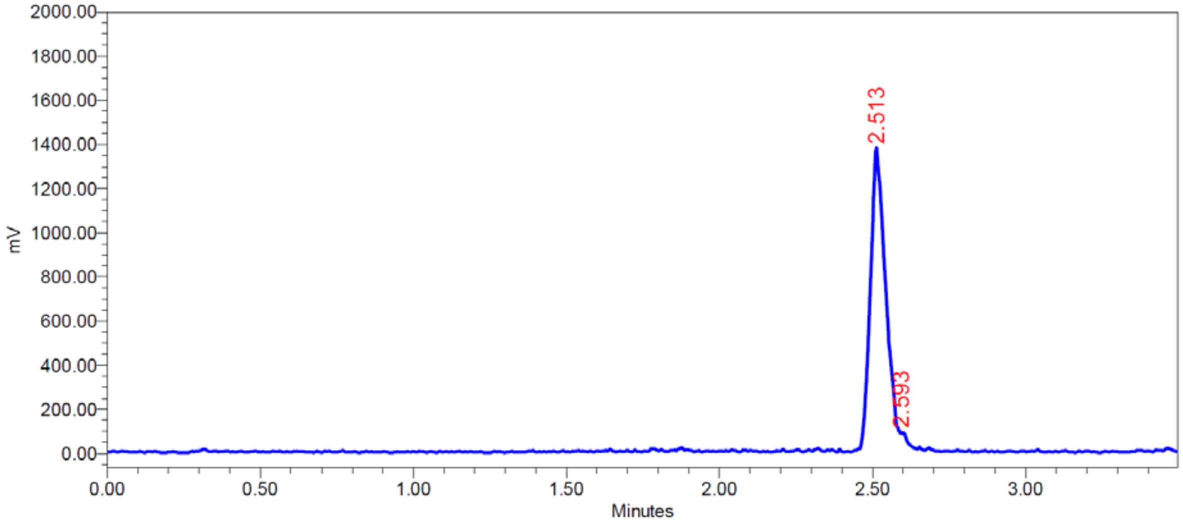

C)

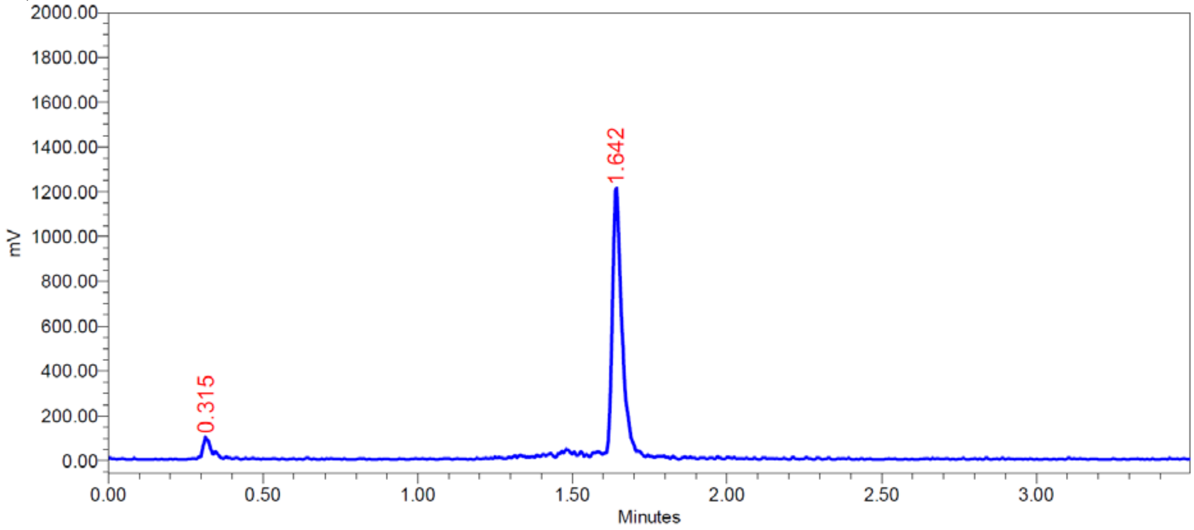

D)

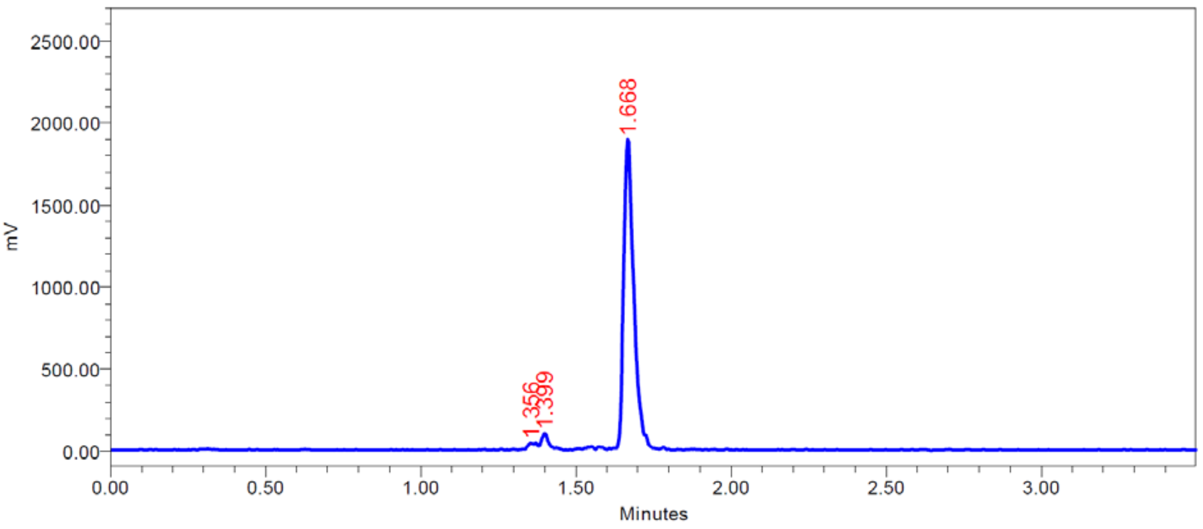

Figure S6: Radio-HPLC chromatograms of [^{203}Pb]Pb-DOTAM-TATE (**A**), [^{203}Pb]Pb-eSOMA-01 (**B**), [^{203}Pb]Pb-eSOMA-02 (**C**) and [^{203}Pb]Pb-eSOMA-03 (**D**).

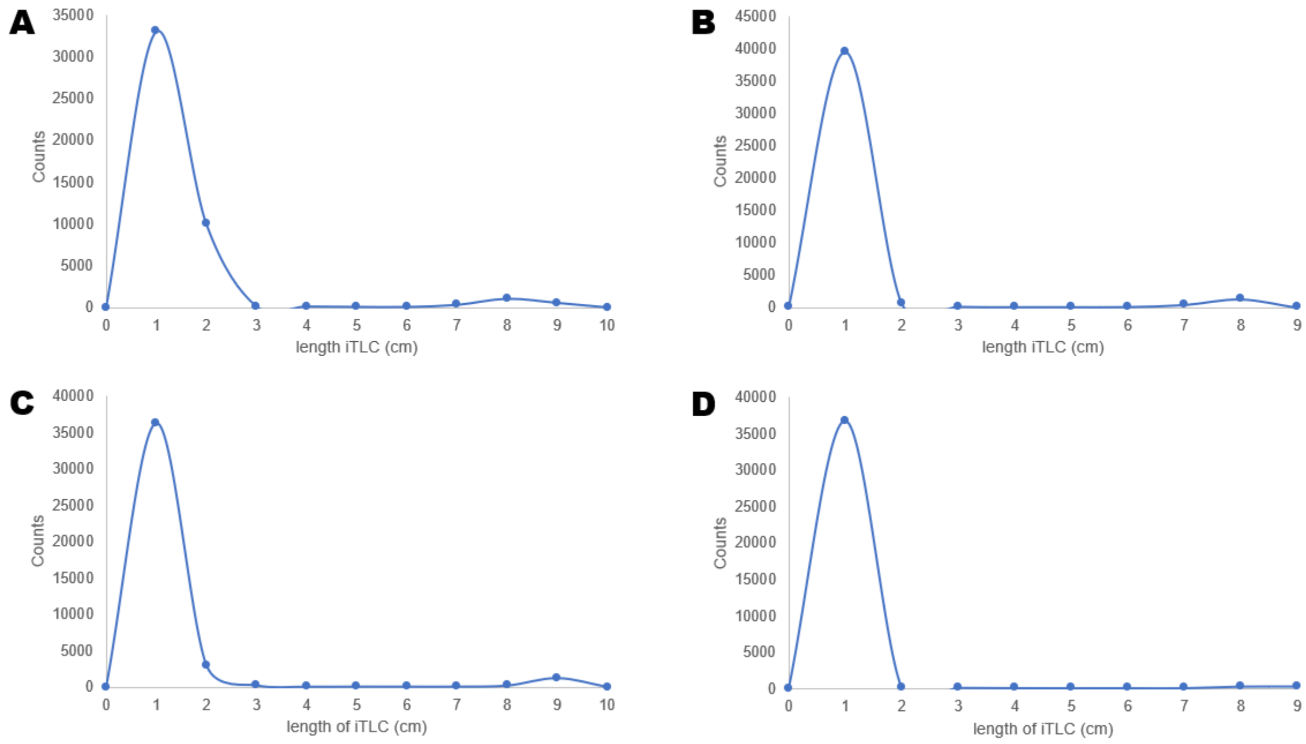

Figure S7: iTLC spectra of [^{212}Pb]Pb-DOTAM-TATE (**A**), [^{212}Pb]Pb-eSOMA-01 (**B**), [^{212}Pb]Pb-eSOMA-02 (**C**) and [^{212}Pb]Pb-eSOMA-03 (**D**).

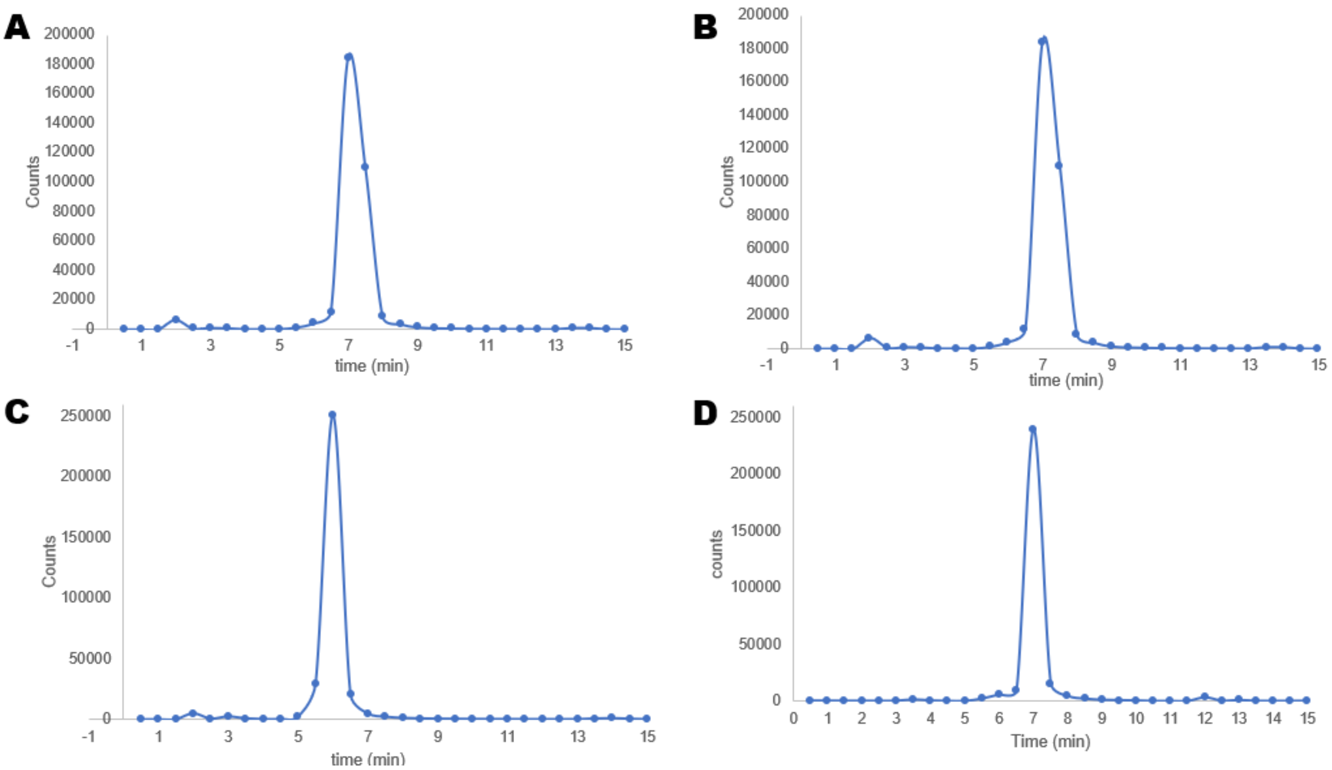

Figure S8: Radio-HPLC chromatograms of [^{212}Pb]Pb-DOTAM-TATE (**A**), [^{212}Pb]Pb-eSOMA-01 (**B**), [^{212}Pb]Pb-eSOMA-02 (**C**) and [^{212}Pb]Pb-eSOMA-03 (**D**).

Table S1: Ex vivo biodistribution analysis at 24 h post-injection (n = 4 mice/compound). Data is represented as percentage of injected dose per gram of tissue (% ID/g).

Tissues	[203Pb]Pb-eSOMA-01	[203Pb]Pb-eSOMA-02
Blood	0.05 ± 0.02	0.17 ± 0.11
Tumor	2.89 ± 0.84	2.54 ± 0.36
Heart	0.05 ± 0.01	0.09 ± 0.02
Lungs	0.36 ± 0.05	0.53 ± 0.04
Liver	0.86 ± 0.10	1.11 ± 0.05
Spleen	0.22 ± 0.03	0.61 ± 0.15
Stomach	0.75 ± 0.34	0.67 ± 0.19
Small Intestine	0.12 ± 0.02	0.24 ± 0.03
Large Intestine	0.34 ± 0.08	0.41 ± 0.06
Pancreas	0.12 ± 0.02	0.83 ± 0.96
Kidneys	7.44 ± 1.17	12.60 ± 0.02
Muscle	0.18 ± 0.03	0.04 ± 0.07
Skin	0.03 ± 0.02	0.43 ± 0.09
Bones	0.27 ± 0.02	0.67 ± 0.04
T/K	0.24 ± 0.18	0.20 ± 0.04

Table S2: Ex vivo biodistribution analysis of [^{212}Pb]Pb-DOTAM-TATE at 1, 4 and 24 h post-injection (n = 4 mice/group). Data is represented as percentage of injected dose per gram of tissue (% ID/g).

Tissues	1 h	4 h	24 h	24 h block
Blood	1.34 ± 0.53	0.02 ± 0.03	-0.06 ± 0.03	-0.02 ± 0.05
Tumor	9.57 ± 3.05	8.04 ± 3.26	6.07 ± 1.28	-0.25 ± 0.04
Heart	0.61 ± 0.34	-0.11 ± 0.03	-0.32 ± 0.04	-0.03 ± 0.18
Lungs	2.75 ± 0.52	0.82 ± 0.36	0.33 ± 0.11	-0.02 ± 0.06
Liver	2.69 ± 0.18	2.26 ± 0.30	1.42 ± 0.17	0.25 ± 0.46
Spleen	0.55 ± 0.21	0.22 ± 0.06	-0.13 ± 0.05	-0.12 ± 0.12
Stomach	2.69 ± 0.53	1.62 ± 0.50	0.61 ± 0.18	0.25 ± 0.28
Intestines	2.03 ± 0.63	0.68 ± 0.08	0.22 ± 0.04	0.00 ± 0.03
Pancreas	5.19 ± 0.73	1.14 ± 0.12	0.28 ± 0.06	-0.11 ± 0.03
Kidneys	53.52 ± 4.18	49.42 ± 2.92	29.20 ± 5.11	25.77 ± 9.18
Muscle	-0.08 ± 0.07	-0.19 ± 0.04	-0.58 ± 0.41	-0.49 ± 0.26
Skin	1.00 ± 0.38	0.59 ± 0.21	-0.83 ± 0.73	-1.22 ± 0.46
Bones	0.69 ± 0.02	0.00 ± 0.04	-0.43 ± 0.16	-0.35 ± 0.2
T/K	0.18 ± 0.08	0.16 ± 0.07	0.20 ± 0.05	-

Table S3: Ex vivo biodistribution analysis of [^{212}Pb]Pb-eSOMA-01 at 1, 4 and 24 h post-injection (n = 4 mice/group). Data is represented as percentage of injected dose per gram of tissue (% ID/g).

Tissues	1 h	4 h	24 h	24 h block
Blood	1.80 ± 0.37	-0.02 ± 0.04	-0.09 ± 0.00	-0.06 ± 0.01
Tumor	16.15 ± 2.31	10.92 ± 3.81	6.83 ± 1.28	-0.45 ± 1.09
Heart	0.71 ± 0.33	-0.02 ± 0.22	-0.41 ± 0.08	-0.20 ± 0.03
Lungs	4.05 ± 0.69	1.38 ± 0.54	0.44 ± 0.20	-0.06 ± 0.05
Liver	4.72 ± 0.53	2.73 ± 0.49	1.70 ± 0.31	1.07 ± 0.14
Spleen	0.98 ± 0.28	0.23 ± 0.12	-0.42 ± 0.20	-0.25 ± 0.13
Stomach	7.08 ± 0.28	2.51 ± 1.51	0.87 ± 0.53	0.05 ± 0.01
Intestines	2.12 ± 0.39	0.97 ± 0.12	0.23 ± 0.02	0.06 ± 0.04
Pancreas	7.86 ± 0.43	1.72 ± 0.16	0.00 ± 0.11	-0.06 ± 0.03
Kidneys	78.83 ± 12.09	39.58 ± 4.95	22.02 ± 4.06	17.81 ± 3.85
Muscle	0.22 ± 0.51	-0.27 ± 0.11	-0.74 ± 0.31	-0.29 ± 0.15
Skin	2.23 ± 0.26	0.17 ± 0.74	-0.82 ± 1.04	-0.64 ± 0.74
Bones	1.75 ± 0.44	0.14 ± 0.09	-0.92 ± 1.19	-1.01 ± 1.45
T/K	0.21 ± 0.03	0.27 ± 0.06	0.33 ± 0.10	-

Table S4: Ex vivo biodistribution analysis of [^{212}Pb]Pb-eSOMA-02 at 1, 4 and 24 h post-injection (n = 4 mice/group). Data is represented as percentage of injected dose per gram of tissue (% ID/g).

**Tissues**	**1 h**	**4 h**	**24 h**	**24 h block**
Blood	1.44 ± 0.24	0.01 ± 0.02	-0.08 ± 0.04	-0.09 ± 0.04
Tumor	7.93 ± 2.14	7.45 ± 0.29	1.02 ± 0.09	-0.40 ± 0.50
Heart	0.54 ± 0.21	-0.07± 0.09	-0.26 ± 0.03	-0.18 ± 0.05
Lungs	3.49 ± 0.70	0.99 ± 0.24	0.20 ± 0.21	-0.09 ± 0.09
Liver	3.96 ± 0.37	3.09 ± 0.70	1.44 ± 0.94	0.83 ± 0.87
Spleen	0.59 ± 0.06	0.39 ± 0.27	-0.08 ± 0.08	-0.01 ± 0.26
Stomach	3.74 ± 0.66	1.17 ± 0.35	0.36 ± 0.27	0.14 ± 0.28
Intestines	2.24 ± 1.05	0.67 ± 0.21	0.13 ± 0.09	0.03 ± 0.03
Pancreas	6.41 ± 0.70	1.25± 0.27	0.41 ± 0.37	-0.02 ± 0.10
Kidneys	81.13 ± 14.27	50.50 ± 3.24	28.22 ± 9.41	37.92 ± 9.17
Muscle	-0.41 ± 0.24	-0.19 ± 0.09	-0.47 ± 0.21	-0.17 ± 0.23
Skin	-0.22 ± 2.40	0.25 ± 0.33	-1.19 ± 0.81	-0.68 ± 0.52
Bones	-1.10 ± 2.05	0.03 ± 0.05	-0.71 ± 0.88	-0.21 ± 0.01
T/K	0.10 ± 0.04	0.12 ± 0.02	0.08 ± 0.06	-
